# Location and Setting of the Mars InSight Lander, Instruments, and Landing Site

**DOI:** 10.1029/2020EA001248

**Published:** 2020-10-08

**Authors:** M. Golombek, N. Williams, N. H. Warner, T. Parker, M. G. Williams, I. Daubar, F. Calef, J. Grant, P. Bailey, H. Abarca, R. Deen, N. Ruoff, J. Maki, A. McEwen, N. Baugh, K. Block, L. Tamppari, J. Call, J. Ladewig, A. Stoltz, W. A. Weems, L. Mora‐Sotomayor, J. Torres, M. Johnson, T. Kennedy, E. Sklyanskiy

**Affiliations:** ^1^ Jet Propulsion Laboratory California Institute of Technology Pasadena CA USA; ^2^ Department of Geological Sciences SUNY Geneseo Geneseo NY USA; ^3^ Department of Earth, Environmental, and Planetary Sciences Brown University Providence RI USA; ^4^ Smithsonian Institution, National Air and Space Museum Washington DC USA; ^5^ Lunar and Planetary Laboratory University of Arizona Tucson AZ USA; ^6^ Lockheed Martin Co. Littleton CO USA; ^7^ Centro de Astrobiología (CSIC/INTA) Instituto Nacional de Técnica Aeroespacial Madrid Spain

**Keywords:** Mars, Mars lander, location, InSight, surface location

## Abstract

Knowing precisely where a spacecraft lands on Mars is important for understanding the regional and local context, setting, and the offset between the inertial and cartographic frames. For the InSight spacecraft, the payload of geophysical and environmental sensors also particularly benefits from knowing exactly where the instruments are located. A ~30 cm/pixel image acquired from orbit after landing clearly resolves the lander and the large circular solar panels. This image was carefully georeferenced to a hierarchically generated and coregistered set of decreasing resolution orthoimages and digital elevation models to the established positive east, planetocentric coordinate system. The lander is located at 4.502384°N, 135.623447°E at an elevation of −2,613.426 m with respect to the geoid in Elysium Planitia. Instrument locations (and the magnetometer orientation) are derived by transforming from Instrument Deployment Arm, spacecraft mechanical, and site frames into the cartographic frame. A viewshed created from 1.5 m above the lander and the high‐resolution orbital digital elevation model shows the lander is on a shallow regional slope down to the east that reveals crater rims on the east horizon ~400 m and 2.4 km away. A slope up to the north limits the horizon to about 50 m away where three rocks and an eolian bedform are visible on the rim of a degraded crater rim. Azimuths to rocks and craters identified in both surface panoramas and high‐resolution orbital images reveal that north in the site frame and the cartographic frame are the same (within 1°).

## Key Points

- A carefully georeferenced high‐resolution image of the InSight lander shows it is located at 4.5024N, 135.6234E in Elysium Planitia, Mars
- Instrument locations are derived by transforming from spacecraft and site frames into the cartographic frame
- A viewshed shows the lander is on a shallow regional slope down to the east and a local slope up to the north

## 1 Introduction

The Interior Exploration using Seismic Investigations, Geodesy and Heat Transport (InSight) spacecraft landed successfully in western Elysium Planitia on Mars on 26 November 2018 (Banerdt et al., 2020). The lander carries a payload focused primarily on exploring the interior of the planet, including the Seismic Experiment for Internal Structure (SEIS) seismometer (Lognonné et al., 2019), the Heat Flow and Physical Properties Package (HP^3^) (Spohn et al., 2018), and the Rotation and Interior Structure Experiment (RISE) (Folkner et al., 2018). The lander also carries an Auxiliary Payload Sensor Suite (APSS) to identify and characterize SEIS detections that are not marsquakes that includes a magnetometer, an atmospheric pressure sensor, and a pair of wind and air temperature sensors (Banfield et al., 2019). The SEIS and APSS instruments are designed to operate continuously, so with imaging of the local area, they can characterize eolian changes. In addition, the lander carries two color cameras, the lander mounted Instrument Context Camera (ICC) and the arm‐mounted Instrument Deployment Camera (IDC) (Maki et al., 2018). The IDC is attached to the forearm of a four degree of freedom Instrument Deployment Arm (IDA), used to deploy the instruments onto the surface, which also includes a scoop at the end that can interact with surface materials (Trebi‐Ollennu et al., 2018). Since landing, a large number of color surface images, mosaics, and panoramas have been acquired including: the lander, its footpads and underlying terrain, three complete stereo panoramas (morning, afternoon, and evening), and stereo coverage at two resolutions (0.5 and 2 mm per elevation posting) of the instrument deployment workspace to select the locations to place the instruments (Golombek et al., 2020).

In general, determining where a spacecraft lands on another planet is important for: understanding the context and setting of the area seen from the lander, planning the traverses and destinations of roving vehicles, relating observations from the surface to those from orbit, and constraining the cartographic‐inertial frame offset. Prior to the advent of high‐resolution imaging that can actually resolve a spacecraft on the surface of Mars, surface panoramas from landers were used to triangulate to common features observed in lower resolution orbital images (e.g., Golombek et al., 1997, 1999; Morris et al., 1978; Morris & Jones, 1980). With the advent of high‐resolution orbital imaging of Mars, especially the High‐Resolution Imaging Science Experiment (HiRISE) camera (McEwen et al., 2007) capable of ~25 cm/pixel images, all of the landed spacecraft have been imaged soon after landing (e.g., McEwen et al., 2010; Parker et al., 2007). In addition, HiRISE has acquired fairly regular images of rovers as they traverse for updates on spacecraft position and the local environment (e.g., Parker et al., 2010, 2012). For the InSight lander, interpretations of the geology of the landing site have relied heavily on its location and setting as determined by HiRISE (Golombek, Warner, et al., 2020).

Understanding InSight's location and setting carries additional importance because of its particular payload and science objectives. SEIS measures accelerations that travel through the planet, so knowing the cartographic location and orientation of the instrument is essential for determining the source region for each marsquake (Giardini et al., 2020). In addition, seismic accelerations travel through the subsurface to be recorded by SEIS, so understanding the elastic and physical properties of these materials is important for their interpretation (Lognonné et al., 2019, 2020). Passive SEIS monitoring of atmospheric disturbances also yields information on the subsurface properties (Golombek et al., 2018; Lognonné et al., 2020), and the spacecraft carries a mole (part of HP^3^), designed to percussively penetrate up to 5 m through unconsolidated material (Spohn et al., 2018) while SEIS records the hammering. This allows the direct measurement of *P* and *S* wave velocities and elastic properties (Kedar et al., 2017; Lognonné et al., 2020). Prior to landing, substantial effort went into characterizing surface materials and the shallow subsurface structure of the region so the mole would be able to penetrate, as part of the landing site selection effort (Golombek et al., 2017, 2018). These orbital investigations interpreted the smooth plains within the 130 km long landing ellipse as being composed of Early Amazonian‐Hesperian lava flows that are about 200 m thick topped by an impact fragmented, relatively fine‐grained regolith mostly 3–5 m thick (Golombek et al., 2017, 2018; Warner et al., 2017). Furthermore, the thickness of the regolith was mapped across the landing ellipse by estimating the source depth of crater ejecta with and without rocks (rocky and nonrocky ejecta craters) 30–60 m in diameter (Golombek et al., 2017, 2018, 2020; Warner et al., 2017). Understanding the true landed location of the spacecraft within that region provides important information about the expected subsurface structure at that specific location, which can be used to aid InSight geophysical interpretations.

Determining where a spacecraft is located on the surface of a planet is also important for future landings. Spacecraft are flown and tracked between planets in an inertial reference frame. Surface features and maps of Mars are in a cartographic frame defined by the planet's radius, equator and a longitude defined as 0°. Landing a spacecraft on Mars requires translating between the inertial frame used for flying and tracking a spacecraft during the transfer trajectory between the planets and the final trajectory correction maneuvers to target an ellipse on the surface of the planet that is mapped in cartographic space. On Mars, previous landers that have been tracked from Earth using radio communications have been located on the surface in inertial space have been used as important fiducials for tying together the two frames (e.g., Golombek et al., 1999). RISE on InSight is expected to determine the location of the lander in inertial space to about five times better than any previous lander on Mars (Folkner et al., 2018), so if it can be accurately located in cartographic space, it will become the best‐known control point on the planet, which could be used to more accurately tie the inertial and cartographic frames together (e.g., Kuchynka et al., 2014).

Determining the location of instruments on the InSight lander is also important for interpreting the data returned. The magnetometer measures both the intensity and the direction of the magnetic field, so understanding its position and orientation on the spacecraft (under the deck) as well as on Mars is important for interpreting the data collected (Johnson et al., 2020). The pressure sensor is housed inside the spacecraft and is connected to the outside atmosphere by a port on the deck of the spacecraft (Banfield et al., 2019). Atmospheric pressure on Mars is directly related to the elevation (Smith & Zuber, 1998), so knowing the elevation of the spacecraft is important for interpreting that data. The wind and temperature sensors are mounted on two masts mounted on the deck. Previous meteorology data from Mars shows that both wind and temperature can vary dramatically with elevation (Schofield et al., 1997), so knowing their height above the surface is important. Meteorology data is also directly affected by weather that is driven by global and regional circulation patterns (Banfield et al., 2020), so knowing where the spacecraft is on Mars geographically is needed to interpret the data.

This paper provides detailed descriptions of where InSight landed, the local setting and what can be seen from the lander, and the locations of all of the instruments on the spacecraft as well as those deployed on the surface of Mars. We begin with the efforts to image the lander on the surface in high‐resolution orbital images and to place the lander into the cartographic frame on Mars using hierarchically georeferenced base maps. Next, we describe the process used to place the instruments on the surface and the frame transformations between spacecraft mechanical, IDA, site frames, and cartographic frames. We then use these transformations to determine the locations in the cartographic frame on Mars of SEIS, HP^3^, the magnetometer, the RISE antennas, the pressure sensor and inlet port, and meteorology masts with their temperature and wind sensors. Using the location of the spacecraft and a HiRISE digital elevation model (DEM), we calculate the viewshed to describe what should be visible from the lander and compare features seen from both the lander and orbital high‐resolution images. We also describe features (rocks and craters) within the panorama from the lander. This work provides the basis for subsequent interpretations of the instrument data that have been returned and will continue to be returned from the InSight lander.

## 2 HiRISE Location of the InSight Lander

The most unambiguous way to determine the location of a spacecraft on the surface of Mars is to acquire a high‐resolution image that shows the lander with respect to other surface features. HiRISE (McEwen et al., 2007) has acquired images that show all previous successful Mars landers and rovers including: Viking landers 1 and 2, Mars Pathfinder, Mars Exploration Rover Spirit and Opportunity, Phoenix lander, and Mars Science Laboratory Curiosity rover (e.g., McEwen et al., 2010; Parker et al., 2007). Images acquired of the latter two spacecraft soon after landing reveal dark spots where the spacecraft and other hardware have removed bright dust. These dark spots slowly brightened with time as dust settled back onto the surface from the atmosphere (Daubar et al., 2015, 2016). Determining the location of a rover as soon as possible after landing substantially aids in planning subsequent traverses (e.g., Arvidson, Anderson, Bartlett, Bell, Blaney, et al., 2004; Arvidson, Anderson, Bartlett, Bell, Christensen, et al., 2004). For the stationary InSight lander, acquiring an image as soon as possible after landing was important for understanding the surface alteration during landing (and the condition of the spacecraft in the event of a mishap), characterizing the amount of dust at the landing site, estimating the thermal effects (cooling) from the lower albedo for HP^3^ thermal modeling, and providing a baseline for subsequent eolian change detection (Golombek et al., 2018; Golombek, Warner, et al., 2020).

**Figure 1:**
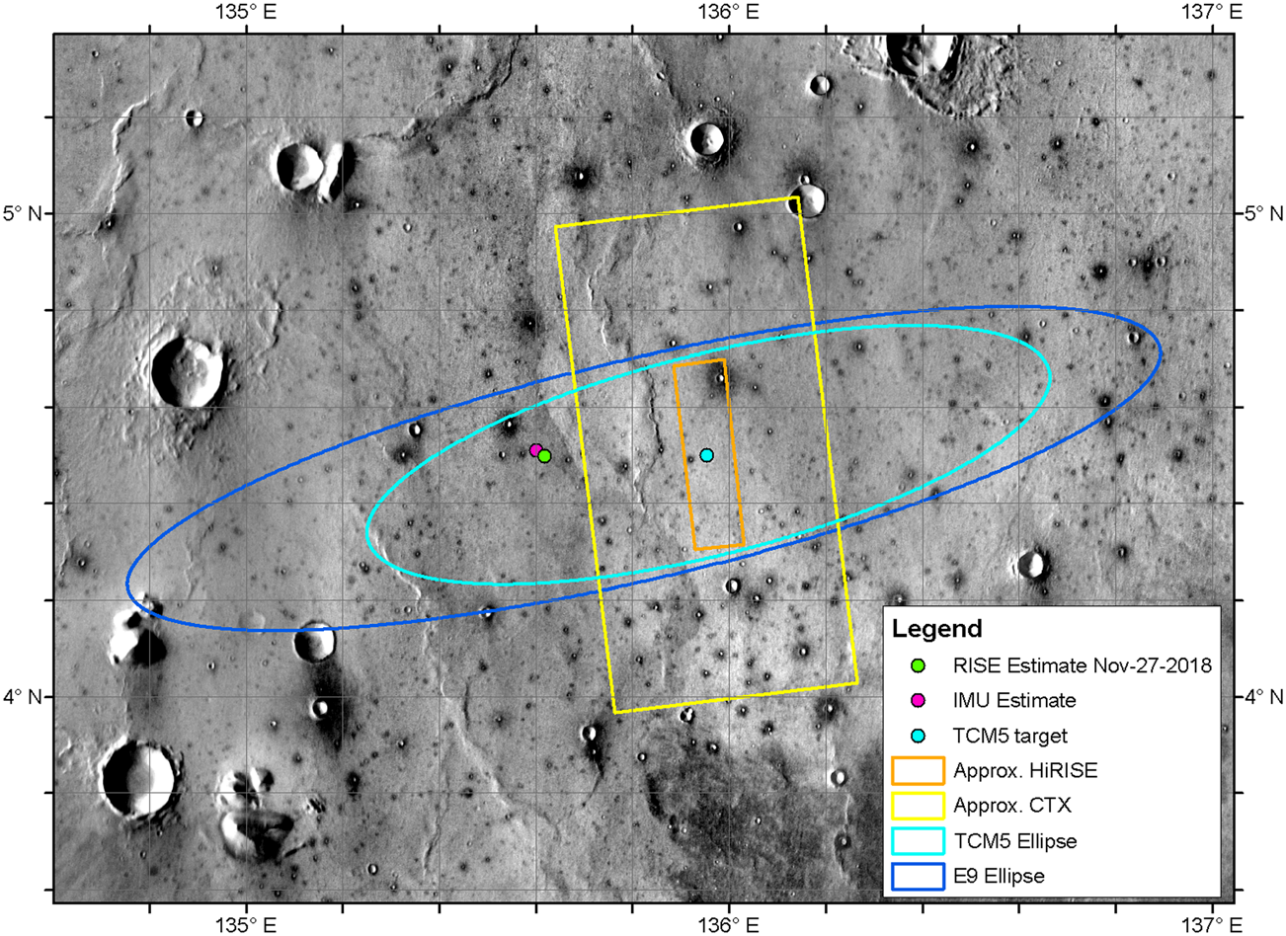
Image showing the reference landing ellipse (E9) in dark blue, the TCM‐5 target point and ellipse in light blue, and the outlines of the HiRISE and CTX images acquired after landing on 29 November 2018. The images did not include the lander, which is near the RISE and IMU estimates to the west. Background image mosaic is from the daytime Thermal Emission Imaging System (THEMIS) infrared global mosaic at 100 m/pixel.

**Figure 2:**
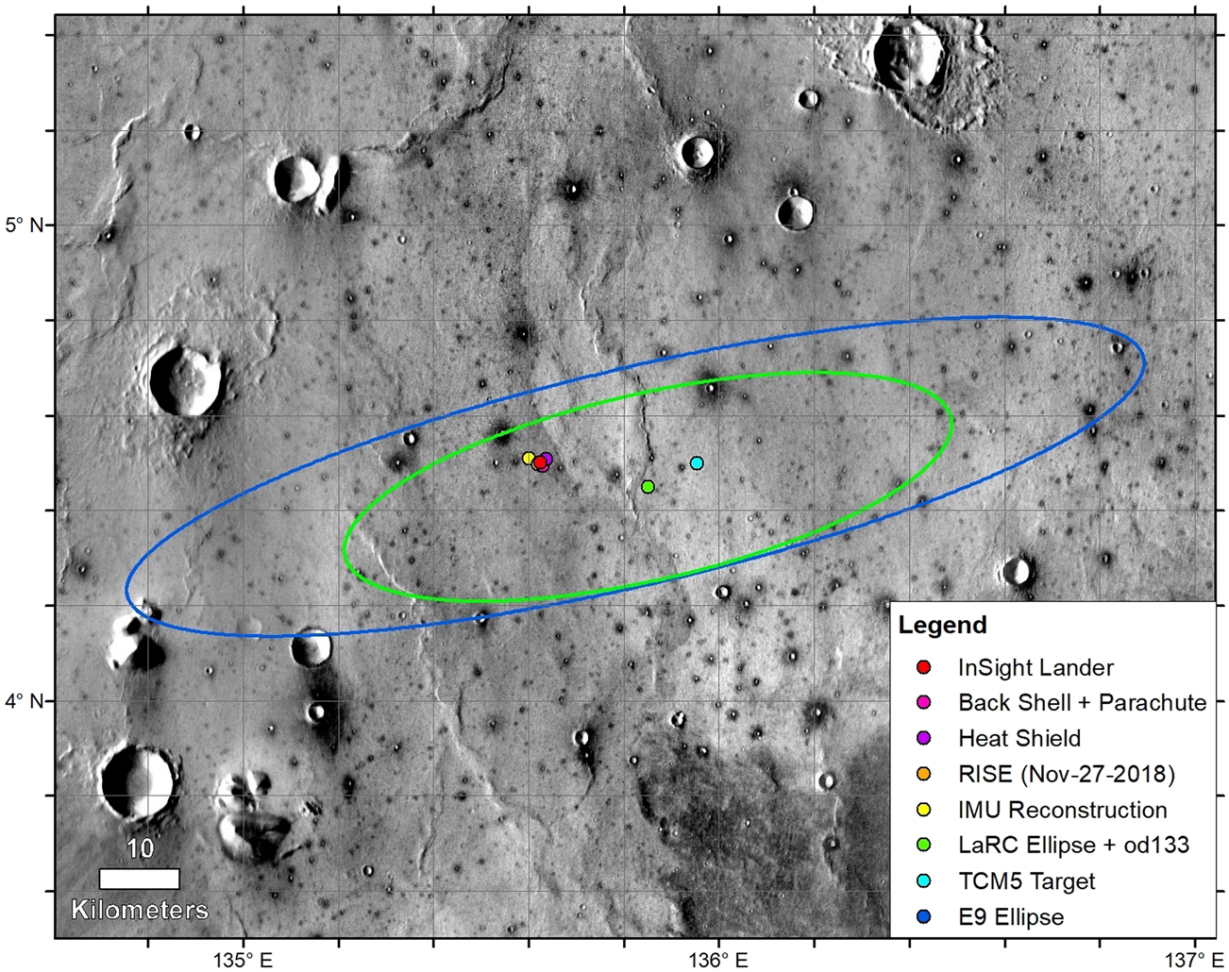
The reference E9 landing ellipse (dark blue, 130 km × 27 km) (Golombek et al., 2017), with trajectory correction maneuver 5 (TCM5) course adjusted target, the last orbit determination solution and ellipse (LaRC green, 77.4 km × 23.2 km), the extrapolated IMU surface location, the RISE estimate from Sol 1 (4.49751° ± 0.00471°N, 135.6178693° ± 0.000337°E), and HiRISE‐based locations on 6 December 2018. Background image mosaic is from the daytime THEMIS infrared global mosaic at 100 m/pixel. The dominant surface is smooth Early Amazonian‐Hesperian plains deformed by north‐trending wrinkle ridges (suggesting subsurface basalt flows) with large impact craters (Golombek et al., 2017, 2018). Most craters larger than around 40 m but smaller than around 2 km are dark (indicating colder daytime temperatures and higher thermal inertia), rocky ejecta craters (Warner et al., 2017). These craters excavate strong coherent rock (basalt) from depths of 4–200 m depth, with a fractured regolith on top and weaker sediments beneath (Golombek et al., 2017, 2018).

Acquiring an image from an orbiter immediately after landing requires careful coordination between the two projects. Well before landing, members of the Mars Reconnaissance Orbiter (MRO) team, the HiRISE team, and the InSight team reviewed the orbits and timing of when possible landing locations would be visible from MRO orbits that passed over the landing ellipse in the time immediately after landing. There were three opportunities to image the landing ellipse in the first few weeks. The first opportunity, 3 days after landing on 29 November 2018, required specifying an exact imaging target location before InSight landed. Spacecraft tracking before the fifth Trajectory Correction Maneuver (TCM‐5) indicated that final maneuver was required because landing was predicted to occur well northeast of the reference landing ellipse, so the center point of the newly predicted landing ellipse that would apply after the TCM‐5 executed was provided as the first imaging location. That location, however, was well east of the final landing location, so the HiRISE image and the colocated Context Camera (CTX; Malin et al., 2007) image acquired did not include the lander (Figure 1). Analysis of tracking data after TCM‐5 indicated that landing was slightly northeast of the preferred target, so TCM‐6 was performed on 25 November. The last orbit determination by the tracking team showed the ellipse center had shifted west‐southwest slightly from the previous solution, but the ellipse had gotten slightly smaller and was now totally within the reference landing ellipse (Figure 2).

**Figure 3:**
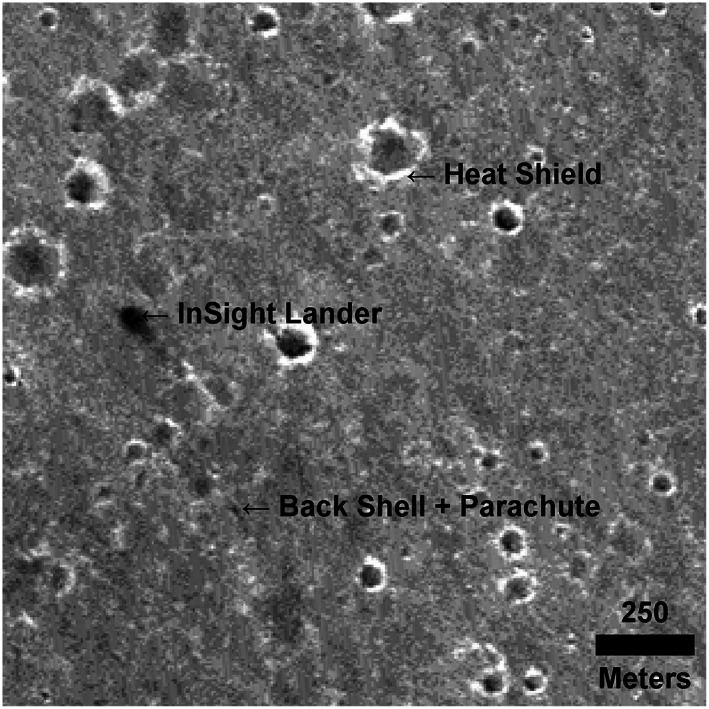
Portion of CTX image K12_057939_1845_XI_04N224W acquired on 6 December 2018 showing the InSight lander, backshell and parachute, and heatshield (with north up).

**Figure 4:**
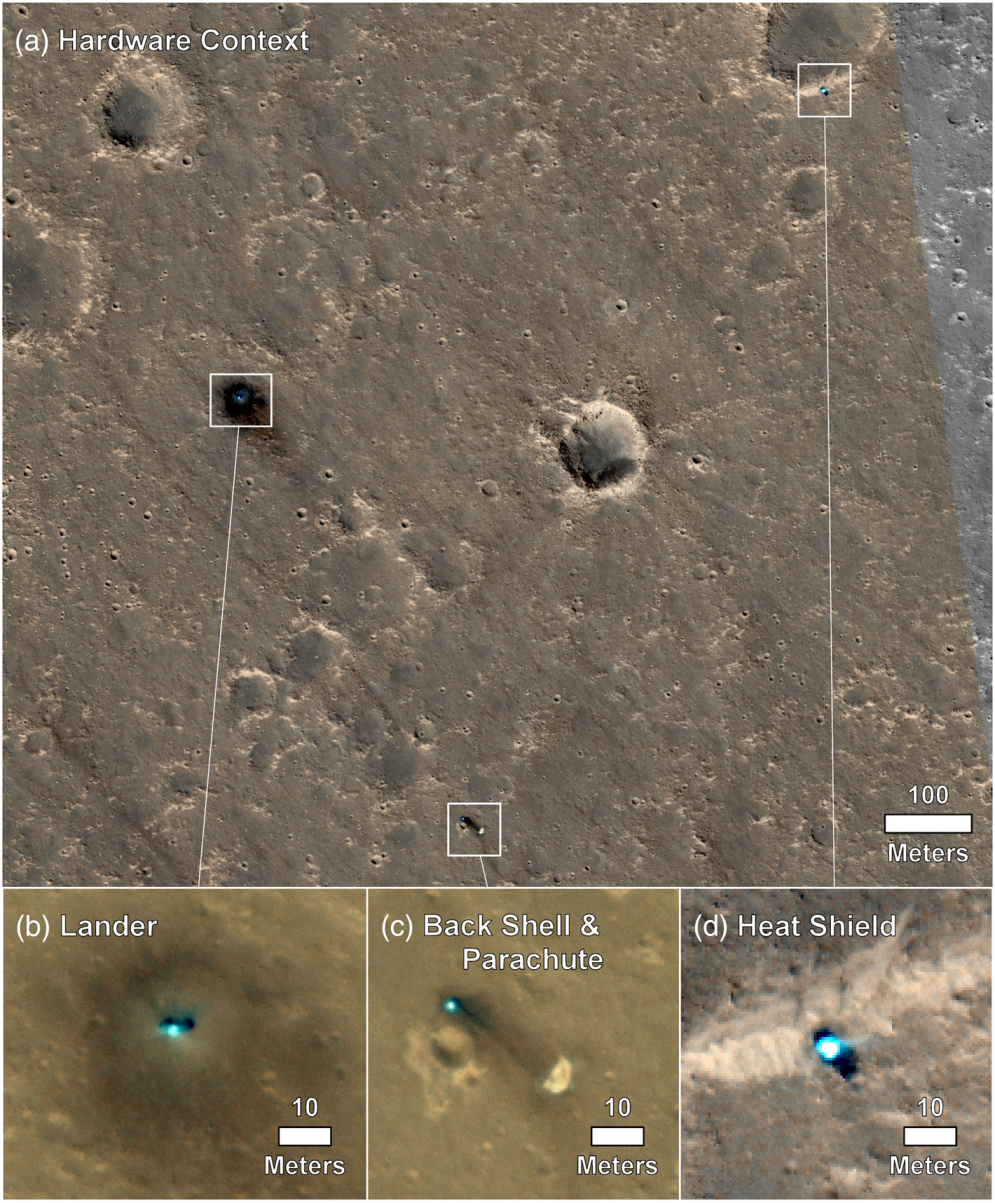
HiRISE images of the InSight lander (and solar panels to either side), heatshield, and parachute/backshell at ~25 cm/pixel (a). Also shown are close ups of the lander (b), parachute and backshell (c), and heat shield (d), in color. Note the dark spots around the lander, the backshell/parachute and heatshield. (a) The hardware context and (d) the heatshield are HiRISE image ESP_058005_1845_RGB acquired on 11 December 2018; (b and c)ESP_057939_1845_RGB (NOMAP) acquired on 6 December 2018.

After successfully landing on 26 November 2018, two estimates of the location of the lander were made (Figure 2). The first was from the RISE tracking solution using the first full day of radio tracking and the second was from the inertial measurement unit (IMU) on the spacecraft, which used accelerometers and gyroscopes to track the position of the spacecraft during atmospheric entry, descent, and landing (Golombek, Warner, et al., 2020). The image target location from RISE, which was expected to be more accurate, was provided to MRO and the HiRISE team on 29 November 2018 for the next image to be acquired on 6 December. The 6 December 2018 HiRISE and CTX images successfully captured the lander, backshell/parachute, and heatshield. The central color HiRISE strip included both the lander and backshell/parachute. The HiRISE team slightly adjusted the camera pointing for the next image on 11 December 2018, to include the heatshield in color. The portions of the CTX and HiRISE images showing the lander and entry and descent hardware acquired on 6 and 11 December 2018 are shown in Figures 3 and 4, respectively.

InSight landed within the 130 km by 27 km reference ellipse selected during project development (Golombek et al., 2017) (Figure 2). As part of landing site selection (Golombek et al., 2017), image and topographic base maps of the region were generated and hierarchically registered to the International Astronomical Union/International Association of Geodesy, IAU/IAG 2000 frame (Archinal et al., 2018; Seidelmann et al., 2002), in the positive East planetocentric coordinate system with 463 m/pixel Mars Orbiter Laser Altimeter (MOLA) elevation postings as its base (Smith et al., 2001). Higher resolution images and DEMs were carefully geo‐referenced in hierarchical order by increasing resolution, starting with 12.5 m/pixel High‐Resolution Stereo Camera (HRSC) images (Gwinner et al., 2016), then tie‐pointing 6 m/pixel MRO Context Camera (CTX) images (Malin et al., 2007) to the HRSC images, and finally tie‐pointing 0.25 m/pixel HiRISE images (McEwen et al., 2007) to the CTX images (Fergason et al., 2017). Georeferencing was done by two groups. Fergason et al. (2017) hierarchically georeferenced HRSC and CTX and de‐jittered HiRISE image DEMs to the MOLA base to produce a combined DEM at 20 m/pixel. HiRISE DEMs were produced at 1 m per elevation posting with an internal precision of 0.2 m and an uncertainty with respect to MOLA of order ~10 m. Golombek et al. (2017) also used orthophotos (geometrically corrected from stereo images), map‐projected images where orthophotos were unavailable and nonmap‐projected HiRISE images were used as the final, “top” level of the hierarchy as they have the highest resolution possible. About 20 to 50 tie points were used to georeference CTX orthophotos and map projected images to the HRSC orthophoto with an estimated spatial accuracy of ±10–20 m. About 20 to 30 tie points were used to georeference HiRISE orthophotos and map projected images to the CTX orthophotos with an estimated spatial accuracy of ±10 m (Golombek et al., 2017).

After landing, both prelanding and postlanding images were reprocessed with higher precision to the HRSC base map (level 4 image HC573_0000; Golombek et al., 2017). Pixel darkening toward the edges of the CTX orthoimage F04_037262_1841_XN_04N224W (Fergason et al., 2017) was normalized by removing a degree 4 polynomial fit of the cross‐track darkening artifact. Then, the CTX orthoimage was coregistered with a single rigid shift to HRSC to align the circular rims and centers of large craters visible in both images to within a single pixel precision (12.5 m). This registration is 16 m from the original registration in Golombek et al. (2017) and ~187 m from that of Fergason et al. (2017). InSight had the good fortune to land in one of seven HiRISE DEMs of the landing ellipse created prior to landing (InSightE17_C; Fergason et al., 2017). The original ESP_036761_1845 HiRISE ortho and DEM were similarly tie‐pointed using craters across the image and used least squares fitting of six tie points to perform an affine transformation (translation, rotation, scale, and skew) without introducing nonlinear distortions to better match the CTX and HRSC to within ~6 m. This registration is 16 m from the original registration by Golombek et al. (2017) and 273 m from that of Fergason et al. (2017). Following this updated geo‐referencing of the orthoimage, which during DEM generation had previously removed viewing geometry distortions for the undulating surface, other post landing HiRISE images were then coregistered to the established orthoimage. Images taken after landing were similarly registered to this same coordinate frame to compensate for HiRISE images' own viewing geometry distortions.

**Figure 5:**
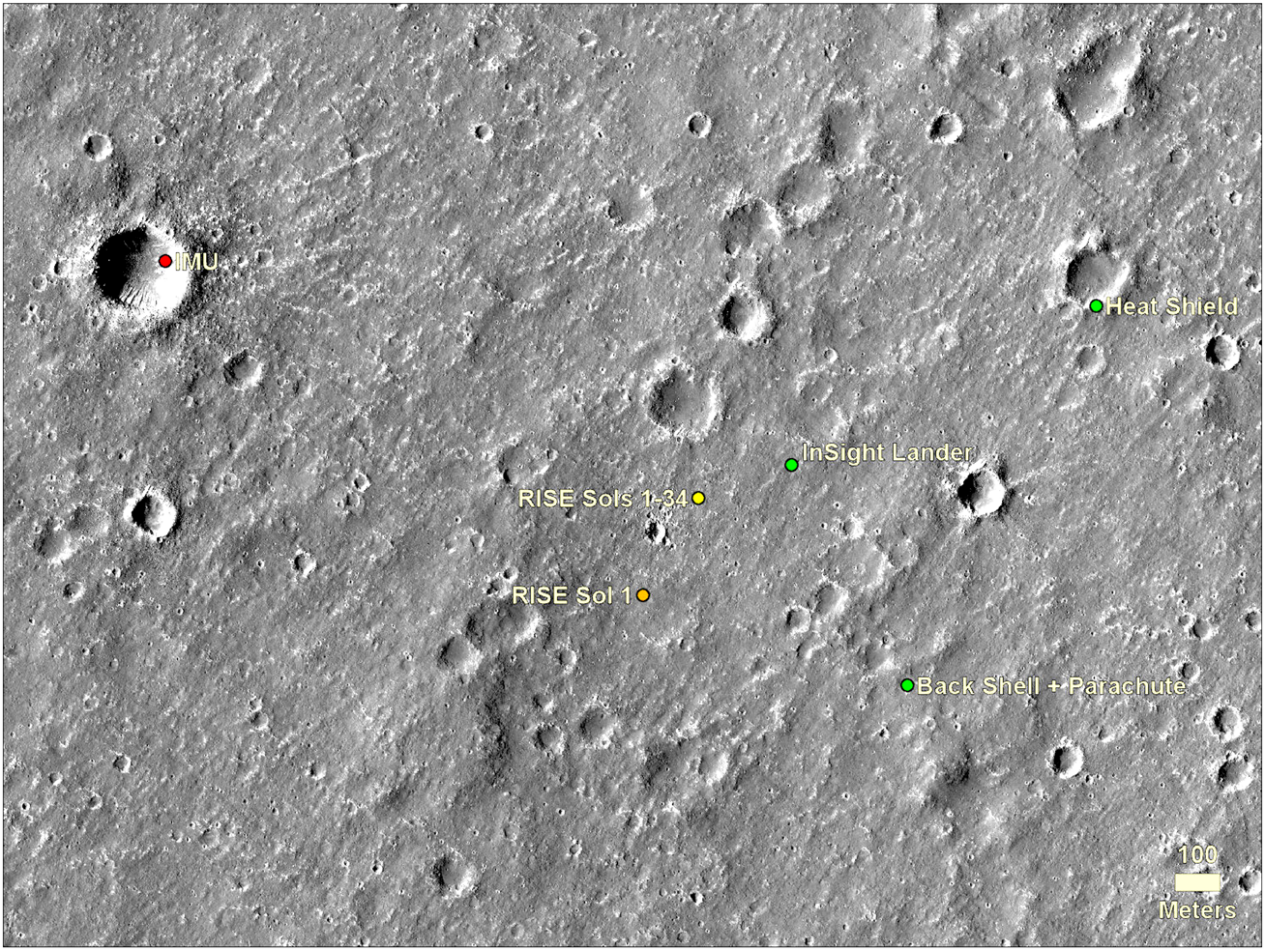
Area around the lander in HiRISE image showing the location of the lander, heatshield, and backshell (and parachute) and estimated positions from the IMU within the first few days after landing and RISE after the first day and after 34 sols of tracking with an improved rotation model (Golombek, Warner, et al., 2020). HiRISE image ESP_036761_1845, which is one of the stereo pairs used for the HiRISE DEM of this area. North is up.

**Figure 6:**
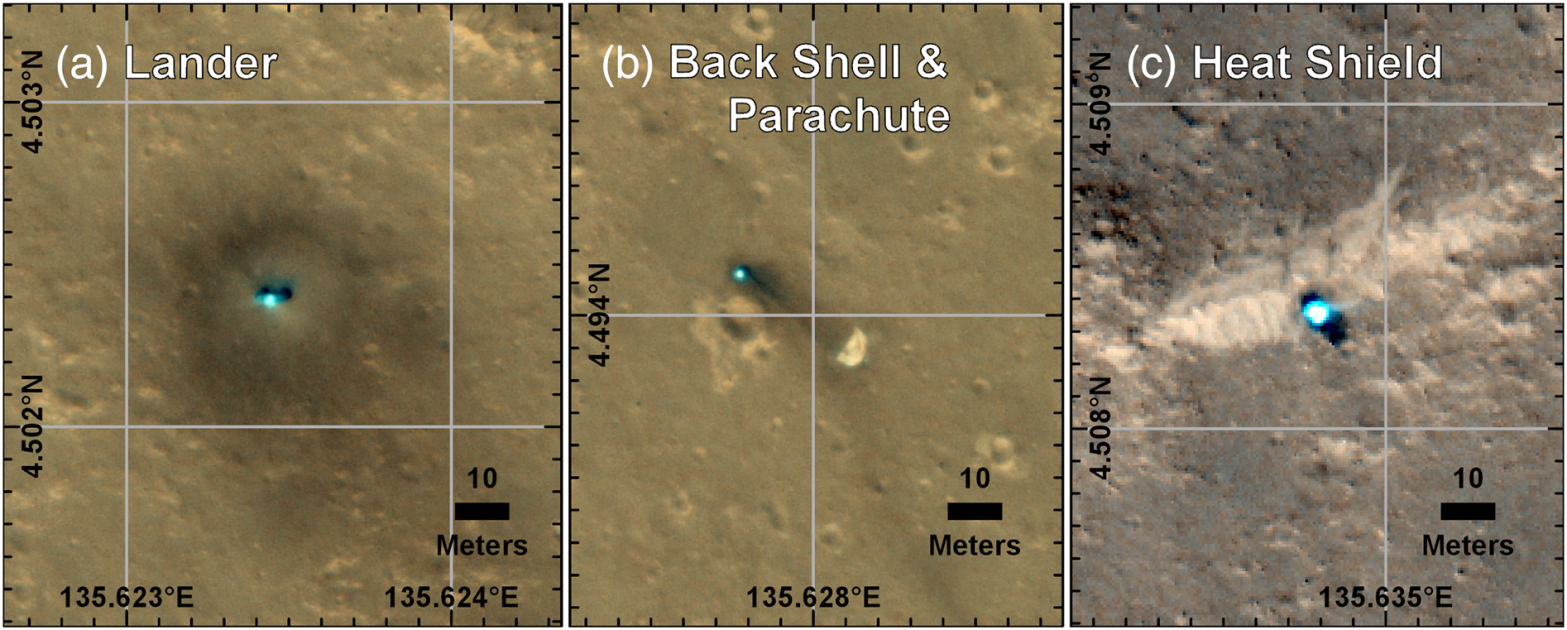
Color HiRISE images of the InSight Lander (a), and backshell/parachute (b), and heat shield (c), taken 11 December. With incremental grid lines of 0.001° used to determine their locations in cartographic space. HiRISE images ESP_057939_1845_RGB (lander, backshell/parachute) and ESP_058005_1845_RGB (heatshield) georeferenced to ESP_036761_1845_RED orthoimage.

Using the coordinates in the georeferenced HiRISE image acquired on 6 December 2018, the lander is located at 4.50238417°N (northing = 266,877.460 m), 135.62344690°E (easting = 8,039,038.792 m), at an elevation of −2,613.426 m relative to the local Mars geoid radius from MOLA (Smith et al., 2001) of 3,390,460.34 m (Figures 5 and 6) in the northwest‐central portion of the landing ellipse in western Elysium Planitia (Golombek, Warner, et al., 2020; Parker et al., 2019).

The RISE (Folkner et al., 2018) inertial location estimated from X‐band radio tracking from the first 34 sols of the mission is ~220 m west of the HiRISE location (Golombek, Warner, et al., 2020) (Figure 5). This is a measure of the cartographic map tie uncertainty with respect to inertial coordinates in this part of Mars. This uncertainty is similar to previous measurements of this offset with the IAU/IAG 2000 frame (Arvidson, Anderson, Bartlett, Bell, Blaney, et al., 2004; Arvidson, Anderson, Bartlett, Bell, Christensen, et al., 2004), although the direction and size of the offset will vary around the planet. This uncertainty results from a number of contributing factors. Inertial coordinates are converted to cartographic coordinates by a rotation model that includes an arbitrary definition of the zero longitude and the planetary radius. The rotation model was recently updated with RISE measurements (Golombek, Warner, et al., 2020). The current IAU/IAG 2000 cartographic frame is based on the global MOLA elevation model as the base because it defines the radius and shape of Mars (Smith et al., 2001). The MOLA laser altimeter obtained elevations every 300 m along roughly polar orbit tracks and near the equator the tracks are typically spaced several kilometers apart. The resulting global elevation model has a resolution of 463 m per elevation posting and many of the postings near the equator have no measured elevations, requiring interpolation from adjacent pixels where elevations exist. The zero longitude on Mars is defined as the center of the Airy‐0 crater, but this point cannot be defined better than ~100 m in the MOLA data (Duxbury et al., 2014). The Mars Global Surveyor spacecraft on which MOLA operated was tracked from Earth during its orbits and via analysis of crossover orbits was able to correct the MOLA elevation tracks to within ~100 m (Neumann et al., 2001). As a result, the tie between the cartographic position and inertial position is typically between 100 and 300 m, even if the georeferencing of higher resolution map products (e.g., HRSC, CTX, and HiRISE) are perfect. The continued tracking of InSight will improve its inertial location to five times better than previous missions (Folkner et al., 2018), so this location could be used as a new fiducial location for tying the two frames together (e.g., Kuchynka et al., 2014). Although the tie between the cartographic and inertial frames is inherently uncertain by 100–300 m, the location of the instruments with respect to the lander is known to within a centimeter as discussed in section 3. In addition, the location of the spacecraft (lander, heatshield, and backshell) within a HiRISE image is probably accurate to within\ one or two pixels (~25–60 cm).

InSight landed 13.78 km west‐northwest from the last orbit determination (od133) (post TCM, TCM‐6) prior to entry and within the two‐sigma landing ellipse (1.9σ) (Figure 6). The lander is 1.38 km from the surface location indicated by the IMU determined a few days after landing and well within the expected mean positional error of ~3 km. According to HiRISE, the heatshield is located 0.762 km downtrack (northeast) from the lander, at an azimuth of 62.3° (4.508346°E, 135.634845°N, northing = 267,231.038 m, easting = 8,039,715.141 m, and elevation = −2,617.504 m). This position is thought to be within line of sight from the lander, but the heatshield has not been visually identified or resolved in any images taken by the lander (also see section 4). The backshell/parachute is located 0.553 km to the southeast at an azimuth of 152.3° (4.49413°N, 135.627781°E, northing = 266,388.697 m, easting = 8,039,296.003 m, and elevation = −2,614.012 m).

## 3 Location of Instruments

### 3.1 Instrument Site Selection

**Figure 7:**
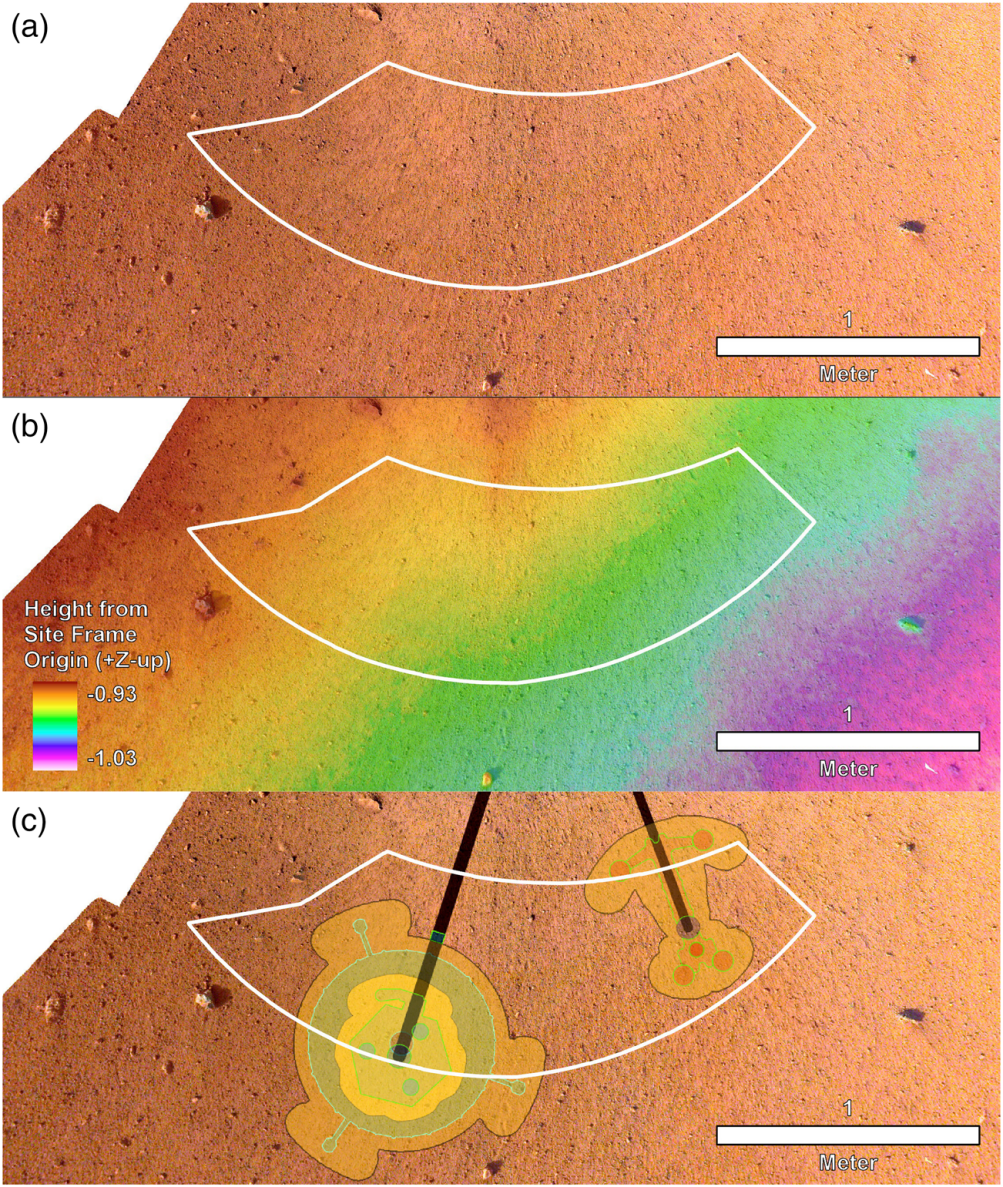
Image mosaic, DEM, and instrument placements selected by the ISSWG and project. (a) The first IDC image mosaic created of the workspace at 1 mm/pixel with the deployment area outlined in white. (b) High‐resolution DEM produced from the second mosaic of the workspace at 2 mm per elevation posting and the deployment area outlined in white. Note that the deployment area has a total relief measured in centimeters. Elevations are reported in negative site frame *Z* coordinates, because site frame has *Z* positive coordinates down, which is opposite of a normal topographic map. (c) Locations selected for the instruments with black lines to the instrument grapple points. SEIS and WTS are to the left and HP^3^ is to the right. North is up for all.

After landing, the highest priority activity after getting the spacecraft in a fully operational configuration was determining where to place the instruments on the surface. The Instrument Site Selection Working Group (ISSWG) was tasked with deciding where to place instruments in the workspace based on the spacecraft tilt, workspace topography, surface characteristics (soils, rocks, etc.) and instrument placement requirements. The ISSWG was composed of six subgroups: (1) geologists, (2) physical property scientists, (3) arm and deployment engineers, (4) Multi‐mission Image Processing Laboratory (MIPL) personnel, and instrument representatives for (5) SEIS and (6) HP^3^. The workspace where the instruments could be placed was in front of the spacecraft (to the south), next to where the arm is attached to the edge of the lander. The workspace is a crescent shaped area that extends out to roughly 2 m away from the lander and 2 m to either side (Figure 7). Instrument placement requirements for SEIS (Lognonné et al., 2019) and HP^3^ (Spohn et al., 2018) are related to surface slope, rocks, load bearing soil, tether geometry, and the desire to be away from the lander (and each other) to reduce noise or interference. Before landing, preliminary preferred instrument locations, with both instruments as far as possible away from the lander and from each other and with SEIS to the west (to avoid crossing tethers), were selected that could be used as starting points for the site selection process.

After landing on 26 November 2018, early ICC images showed dust was on the lens, which made initial workspace mapping difficult. After deployment of the arm and acquisition of IDC images of the spacecraft and solar panels, images of the surrounding terrain were acquired. The first IDC mosaic of the workspace acquired with the arm above the lander was available on 10 December 2018, 2 weeks after landing. Stereo images were processed to create orthoimage and DEM mosaics with 2 mm per elevation posting (Figures 7a and 7b) (Abarca et al., 2019). Analysis of the workspace showed it to be particularly benign with a sandy, granule and pebbly surface, few rocks, and low slopes that met all of the instrument deployment requirements over most of the deployment area. Preliminary instrument locations selected were both near their preferred locations.

A higher resolution workspace stereo mosaic was acquired with the arm below the lander deck and was available on 14 December 2018. Individual frames had a pixel scale of 0.5 mm, and the DEM from these instruments had 1 mm per elevation postings. The preliminary instrument locations met (by a large margin) all instrument deployment requirements in the higher resolution data. The spacecraft testbed with an engineering model lander and arm was sculpted to match the workspace on Mars. The engineering model lander and arm were used to test deployment of the instruments to the selected locations and results showed no problems. The instrument placement locations (Figure 7c) were certified, approved by the instrument Principal Investigators and selected by the project on 17 December 2018.

### 3.2 Frame Transformations

**Figure 8:**
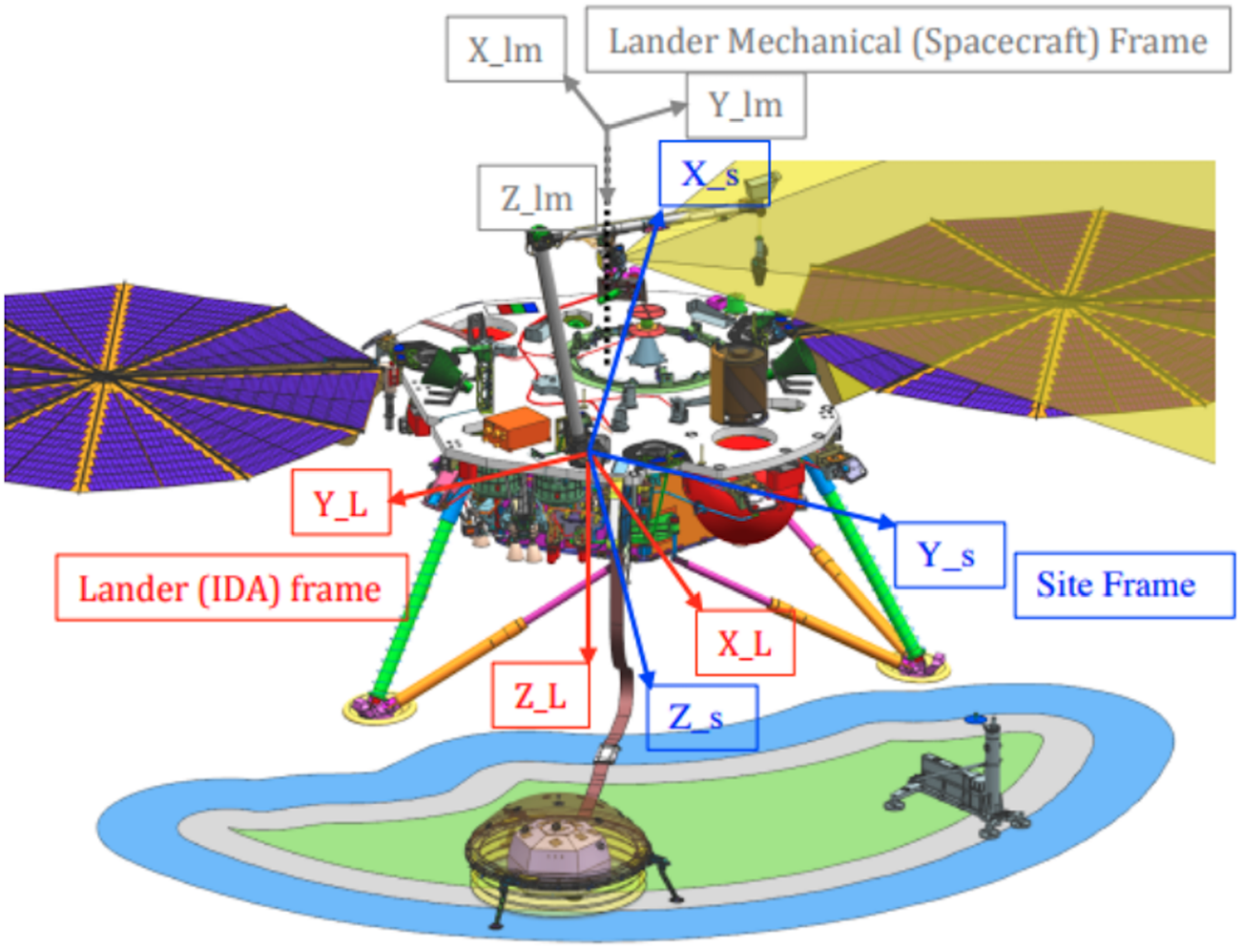
Spacecraft coordinate frames and the deployment space. The lander mechanical (spacecraft) frame (also referred to as the spacecraft mechanical, SM frame in the text) is shown in gray with the origin at the launch vehicle interface about 1 m above the center of the spacecraft deck and the axes marked lm. The lander (IDA) frame and the Site frame have their origins at the base of the shoulder joint of the arm (IDA) on the lander deck. The Lander frame (also referred to as the IDA frame in the text) is in red with the axes marked L. The site frame (referred to as SF in the text) has *Z* down along the gravity vector and is shown in blue with the axes marked s. The deployment space is to the south of the lander and the solar panels are roughly east and west. Note the two RISE antennas (green cones) and the TWINS masts on the edge of the deck in the +*Y* and −*Y* directions of the SM frame. The spacecraft deck is shown in more detail in Figure 10.

The instruments were placed in the workspace in a spacecraft‐centric reference frame, called the IDA frame. To determine their location in the cartographic frame requires a transformation between this frame and others that can be related to their location on Mars. The IDA frame has its origin at the intersection of the IDA azimuth rotation axis (shoulder) and the surface of the lander deck (Figure 8). The IDA frame is fixed relative to the spacecraft mechanical (SM) frame regardless of lander orientation. The SM frame is centered at the launch vehicle interface (about a meter above the center of the deck) with *Z* down perpendicular to the deck, *X* normal to *Z* and perpendicular to a line between the two solar arrays, and *Y* parallel to a line between the two solar arrays in a right handed coordinate frame. The IDA frame is rotated 180° around the +*Z* axis of the spacecraft mechanical frame, and their axes are otherwise always parallel. In SM frame coordinates, the IDA frame origin is located at (−0.775084 m, −0.283362 m, and 0.956056 m). Therefore, the translation of a point in spacecraft SM to IDA frame is defined by the function SM2IDA(argument) = expression (for easier traceability in later equations):

(1) $$\mathrm{SM}2\mathrm{IDA}\left({\left[\begin{array}{c} x\\ y\\ z \end{array}\right]}_{\mathrm{SM}}\right)=\left[\begin{array}{ccc} -1 & 0 & 0\\ 0 & -1 & 0\\ 0 & 0 & 1 \end{array}\right]*\left({\left[\begin{array}{c} x\\ y\\ z \end{array}\right]}_{\mathrm{SM}}-\left[\begin{array}{c} -0.775084\;m\\ -0.283362\;m\\ 0.956056\;m \end{array}\right]\right)={\left[\begin{array}{c} x\\ y\\ z \end{array}\right]}_{\mathrm{IDA}}$$

The site frame has the same origin as the IDA frame but has *Z* along the gravity vector, with *X* north, *Y* east, and *Z* down (right‐hand rule; note that other “elevations” we report are the negative of site frame's Z and this distinction between ±*Z* and elevation are accounted for in the following equations), and is rotated relative to the IDA frame based on postlanding orientation of the vehicle. The site frame thus accounts for the local tilt, expected to be known to <0.6° and orientation of the spacecraft, expected to be known to <0.85° provided by the IMU accelerometers and gyroscopes, respectively. Note the spacecraft is designed to land with +*X* axis of the IDA frame pointing due south, so the workspace is south of the lander. At touchdown, the IMU indicated the IDA X axis was 0.26° counterclockwise of due south. The IDA frame yaw, pitch, and roll angles (rotations about the *Z*, *Y*, and *X* axes, respectively) in site frame are 180.495°, −2.684°, and −2.934° and are very close to expected values of 180°, 0°, and 0° when accounting for the lander tilt of 3.975° in an azimuthal direction of 133.408° in site frame measured clockwise from north (i.e., toward the southeast) or in the IDA frame at an azimuth of 312.913° measured clockwise from the *X* axis toward +*Y* in the *XY* plane. Information needed to transform from the SM and IDA frames to site frame is as follows:

$$\text{Spacecraft Mechanical Frame quaternion}\;\left(\text{scalar last}\right)=\left[\begin{array}{c} 0.02548965\\ 0.02352163\\ 0.00371338\\ 0.99939123 \end{array}\right]$$

$$\mathrm{IDA}\;\text{Frame quaternion}\;\left(\text{scalar last}\right)=\left[\begin{array}{c} 0.02352163\\ -0.02548965\\ 0.99939123\\ -0.00371338 \end{array}\right]$$

$$\mathrm{IDA}\;\text{Frame quaternion}\;\left(\text{scalar first}\right)=\left[\begin{array}{c} -0.00371338\\ 0.02352163\\ -0.02548965\\ 0.99939123 \end{array}\right]$$

$$\text{Gravity vector in}\;\mathrm{IDA}\;\text{frame}=\left[\begin{array}{c} 0.046825\\ -0.051123\\ 0.997594 \end{array}\right]$$

$$\mathrm{IDA}\;\text{frame rotation matrix}\;R\_\mathrm{IDA}=\left[\begin{array}{ccc} -0.99887 & 0.0062231 & 0.047204\\ -0.0086214 & -0.99867 & -0.050774\\ 0.046825 & -0.051123 & 0.99759 \end{array}\right]$$

Note that this IDA frame rotation varies only slightly from the nominal perfectly gravity‐leveled orientation of $\left[\begin{array}{ccc} -1 & 0 & 0\\ 0 & -1 & 0\\ 0 & 0 & 1 \end{array}\right]$. This corresponds to a tilt direction in Site frame (where N = 0°, E = 90°, and W = 270°) of 133.408°.

To convert IDA frame coordinates to site frame, the transformation is defined by the function IDA2Site(argument) = expression:

(2) $$\mathrm{IDA}2\text{Site}\left({\left[\begin{array}{c} x\\ y\\ z \end{array}\right]}_{\mathrm{IDA}}\right)=R\_\mathrm{IDA}\;*\;{\left[\begin{array}{c} x\\ y\\ z \end{array}\right]}_{\mathrm{IDA}}={\left[\begin{array}{c} x\\ y\\ z \end{array}\right]}_{\text{Site}}$$

Figure 8 shows these three coordinate systems.

**Figure 9:**
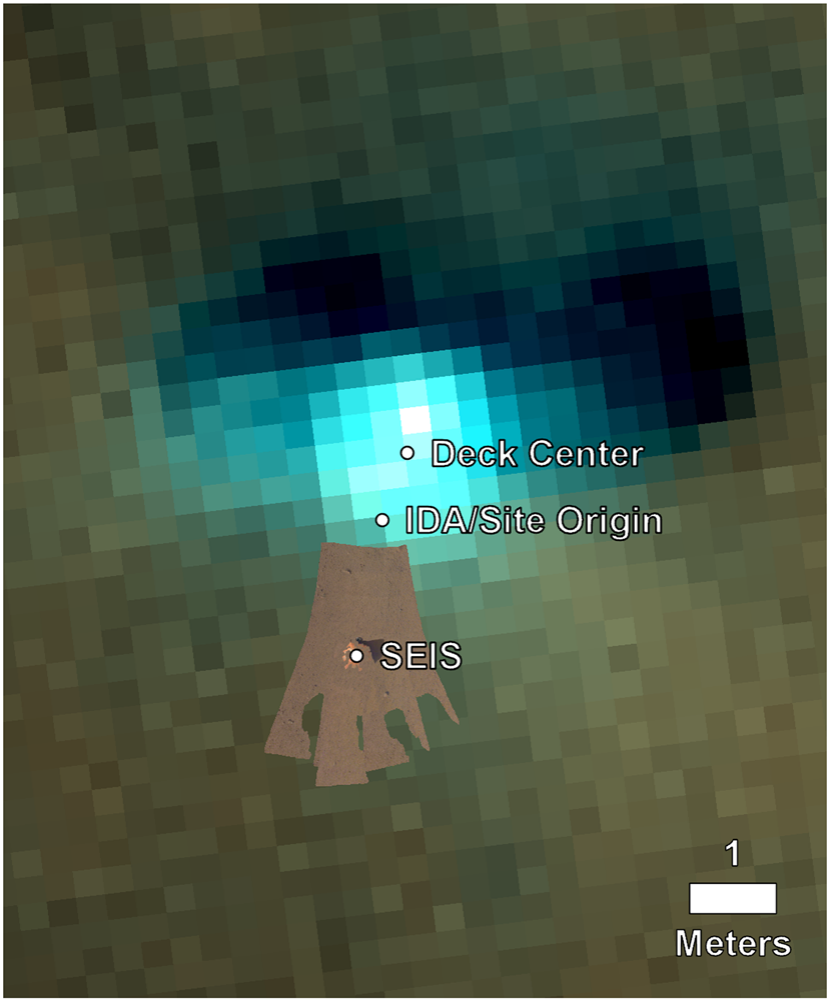
Georeferencing the IDC orthophoto of the workspace from the IDA stereo images and DEM onto the HiRISE image ESP_057939_1845_RGB using the translation from the deck center to the site frame origin. The distance between the center of the spacecraft and the SEIS location is 2.42 m.

The HiRISE image of the lander resolves the lander deck and the solar panels (Figures 4 and 9). The location of the lander determined in the HiRISE image is the center of the lander deck, and the elevation reported is the surface elevation beneath that point along the gravity vector. The location is in the MOLA planetocentric, IAU 2000 cartographic frame, and the elevation is with respect to the MOLA geoid (Smith et al., 2001). The elevation values are from a HiRISE stereo‐derived DEM InSightE17_C, with an internal precision of ~0.2 m (Fergason et al., 2017), which was hierarchically georeferenced to coarser digital elevation models and ultimately the MOLA geoid to of order 10 m using the same tie points as the corresponding orthophoto. Note that the geoid is an equipotential surface derived from the average equatorial radius extended around the planet using the gravity field (Smith et al., 2001) and is measured as a radius from the planet's center of mass. Mars's geoid varies by almost 30 km around the planet, but near the location of the InSight lander has a local radius of 3,390,460.34 m corresponding to an elevation of 0 m for comparison to the elevations described below. Note that the geoid radius is used only for vertical elevations; any horizontal latitude/longitude coordinate transforms use the standard IAU Mars 2000 sphere of uniform radius 3,396,190 m.

In equidistant cylindrical projection using a planetary radius 3,396,190 m, the latitude and longitude values can be converted from degrees to northing and easting in meters by dividing by a unit conversion of:

(3) $$\left[\begin{array}{c} \text{Northing}\;\left(m\right)\\ \text{Easting}\;\left(m\right) \end{array}\right]=\left[\begin{array}{c} \text{Latitude}\;\left({}^{\circ}\right)\\ \text{Longitude}\;\left({}^{\circ}\right) \end{array}\right]*2*\pi *3,396,190\;m/360{}^{\circ}\text{yielding}\;\left[\begin{array}{c} 266,877.460\;m\;N\\ 8,039,038.792\;m\;E \end{array}\right].$$

The inverse for converting northing and easting (in meters) to latitude and longitude (in degrees) is as follows:

(4) $$\left[\begin{array}{c} \text{Latitude}\;\left({}^{\circ}\right)\\ \text{Longitude}\;\left({}^{\circ}\right) \end{array}\right]=\left[\begin{array}{c} \text{Northing}\;\left(m\right)\\ \text{Easting}\;\left(m\right) \end{array}\right]*\left(\frac{360{}^{\circ}}{2*\pi *3,396,190\;m}\right).$$

We define the center of the deck of the spacecraft to be at $\left[\begin{array}{c} 0\;m\\ 0\;m\\ 0.956056\;m\; \end{array}\right]$ in the SM frame. This location has the same latitude and longitude coordinates determined for the center of the spacecraft in the HiRISE image, but the initial elevation relative to MOLA must be calculated. However, unlike site frame, the spacecraft mechanical frame is not horizontal and vertical relative to gravity, so the elevation must first be calculated for a point in the site frame and then transformed via Equations 2 and 1.

To determine the elevation offset of the site frame origin to MOLA, we take a known site frame *Z* value and a known value in a MOLA‐tied HiRISE DEM. The IDA team, using the methods described in section 3.4, determined the ground below the center of the SEIS to be at −1.5733, −0.2955, and 0.9957 m in site frame (transformed from the IDA frame). In addition, we have taken the orthophotos from the workspace DEMs projected in site frame and anchored them on the HiRISE image using the horizontal offset calculated from the center of the lander to the site frame to determine the latitude and longitude of the instruments (Figure 9). The center of SEIS is located at 4.502345°N, 135.623436°E, and the elevation of the ground beneath the center point of SEIS along the *Z* axis of the instrument in the corresponding HiRISE DEM is −2,613.454 m. Assuming this location is the same as that determined by the IDA team yields an elevation offset of +0.9957 m, providing an elevation for the site frame origin of −2,612.4583 m.

### 3.3 Site Frame Origin and Deck Center

With a horizontal tie established at the deck center between site frame and the Mars global frame, the origin of the site frame can then be calculated by combining the deck center horizontal coordinates, elevation from the site frame's offset value determined in section 3.2, and calculating the horizontal offset of the site frame origin from the spacecraft center as follows:

(5) $$\left[\begin{array}{c} 266877.460\;m\\ 8039038.792\\ 0\;m \end{array}\;m\right]+\left[\begin{array}{c} 0\\ 0\\ -2612.4583\;m \end{array}\right]+\left[\begin{array}{ccc} 1 & 0 & 0\\ 0 & 1 & 0\\ 0 & 0 & 0 \end{array}\right]*\mathrm{IDA}2\text{Site}\left(\mathrm{SM}2\mathrm{IDA}\left(\left[\begin{array}{c} 0\\ 0\\ 0.956056\;m \end{array}\right]\right)\right)=\left[\begin{array}{c} 266876.688\;m\\ 8039038.502\;m\\ -2612.458\;m \end{array}\right]$$

Any other coordinates in site frame may now be converted to latitude, longitude, and elevation by adding the site frame *X*, *Y* values to the site frame origin's horizontal coordinates and subtracting the site frame *Z* value from the site frame origin vertical coordinate due to the down→up conversion, as defined by the function Site2LL(argument) = expression:

(6) $$\text{Site}2\mathrm{MF}\left({\left[\begin{array}{c} x\\ y\\ z \end{array}\right]}_{\text{Site}}\right)=\left[\begin{array}{c} 266876.688\;m\\ 8039038.502\;m\\ -2612.458\;m \end{array}\right]+\left[\begin{array}{ccc} 1 & 0 & 0\\ 0 & 1 & 0\\ 0 & 0 & -1 \end{array}\right]*{\left[\begin{array}{c} x\\ y\\ z \end{array}\right]}_{\text{Site}}=\left[\begin{array}{c} \text{Northing}\;\left(m\right)\\ \text{Easting}\;\left(m\right)\\ \text{Elevation}\;\left(m\right) \end{array}\right],$$

where MF refers to the global Mars cartographic frame with coordinates of northing, easting, and elevation from the local geoid.

Similar to calculating the site frame origin, to determine the latitude, longitude, and elevation (with respect to the MOLA geoid) of other instruments rigidly attached to the lander, we must obtain the full three dimensional coordinate of the spacecraft deck center from the horizontal location of the lander as determined by the georeferenced HiRISE and from summing the elevation offset with the difference in elevation from the spacecraft deck center to the site frame origin:

(7) $$\left[\begin{array}{c} 266877.460\;m\\ 8039038.792\;m\\ 0\;m \end{array}\right]+\left[\begin{array}{c} 0\;m\\ 0\;m\\ -2612.4583\;m \end{array}\right]+\left[\begin{array}{ccc} 0 & 0 & 0\\ 0 & 0 & 0\\ 0 & 0 & -1 \end{array}\right]*\mathrm{IDA}2\text{Site}\left(\mathrm{SM}2\mathrm{IDA}\left(\left[\begin{array}{c} 0\;m\\ 0\;m\\ 0.956056\;m\; \end{array}\right]\right)\right)=\left[\begin{array}{c} 266877.460\;m\\ 8039038.792\;m\\ -2612.436\;m \end{array}\right]$$

Using these transformations, the frame origin and deck center locations are shown in Table 2 and may be used to calculate the locations of all other instruments with locations defined in any other frame. We report locations to the millimeter level but believe they are accurate to around 1 cm with respect to the lander location.

### 3.4 SEIS, WTS, and HP^3^ Components' Locations

After placing the instruments in the workspace, several methods were used to determine their actual location by the IDA team. Stereo IDC images of the instruments and their fiducial points were used to determine the instrument locations using stereogrammetry. The commanded position of the arm with the grapple (as a plumb bob) positioned directly above the instrument grapple point was also used. In addition, the workspace orthophotos projected in site frame located on the HiRISE image using the horizontal offset calculated from the center of the lander to the site frame was also used to determine the latitude and longitude of the instruments (Figure 9). Comparison of these two methods to determine the center of the SEIS and its wind and thermal shield (WTS) location is shown in Table 1 and is within 1–2 cm. We also used these same two methods to determine the first location of the HP^3^ support structure (both agreed to within 1–2 cm) (Table 1). The *XYZ* in site frame, the location near the grapple point of the HP^3^, and the ground elevation along the *Z* axis of the instrument are reported in Table 2. The location of the bottom of the cap of the mole on Sol 407, which is near the entrance of the HP^3^ mole into the subsurface (Table 2) is 5 cm above the ground level beneath the first location of HP3 grapple point. Given the tilt of the mole, the latitude and longitude where the mole enters the ground are that of the top cap, and the elevation is that of the ground elevation beneath the grapple point.

**Table 1:** Location of Center of the SEIS in the MOLA Positive East Planetocentric Coordinate System Referenced to the IAU/IAG 2000 Frame Using Two Methods Discussed in the Text

Instrument/object	Method	Latitude (°N)	Longitude (°E)	Northing (m)	Easting (m)
SEIS	IDA Location	4.50234545	135.62343621	266,875.165	8,039,038.159
	Georeferencing	4.50234525	135.62343601	266,875.153	8,039,038.147
WTS	IDA Location	4.50234638	135.62343631	266,875.220	8,039,038.164
	Georeferencing	4.50234611	135.62343622	266,875.204	8,039,038.159

**Table 2:** Location of Spacecraft and Instruments in Spacecraft Mechanical (SM), IDA, and Site (Site) Frames

Instrument	SM *x* (m)	SM *y* (m)	SM *z* (m)	IDA *x* (m)	IDA *y* (m)	IDA *z* (m)	Site *x* (m)	Site *y* (m)	Site *z* (m)	Easting (m)	Northing (m)	Longitude (°N)	Latitude (°E)	Elevation (m)
Ground Under Spacecraft Center	−0.0463	0.0506	1.9432	−0.7287	−0.3339	0.9871	0.7724	0.2897	0.9677	8,039,038.7920	266,877.4600	135.62344690	4.50238417	−2,613.4260
Spacecraft Frame Origin	0.0000	0.0000	0.0000	−0.7751	−0.2834	−0.9561	0.7273	0.3382	−0.9756	8,039,038.8405	266,877.4149	135.62344772	4.50238341	−2,611.4827
Spacecraft Deck Center	0.0000	0.0000	0.9561	−0.7751	−0.2834	0.0000	0.7724	0.2897	−0.0218	8,039,038.7920	266,877.4600	135.62344690	4.50238417	−2,612.4365
IDA and Site Frame Origin	−0.7751	−0.2834	0.9561	0.0000	0.0000	0.0000	0.0000	0.0000	0.0000	8,039,038.5023	266,876.6876	135.62344201	4.50237114	−2,612.4583
SEIS Grapple Hook	−2.3757	−0.5334	1.5812	1.6006	0.2500	0.6251	−1.5677	−0.2952	0.6858	8,039,038.2071	266,875.1199	135.62343703	4.50234469	−2,613.1441
SEIS Ground Location	−2.3958	−0.5178	1.8901	1.6207	0.2344	0.9340	−1.5733	−0.2955	0.9957	8,039,038.2068	266,875.1143	135.62343703	4.50234460	−2,613.4540
WTS Grapple Hook	−2.3158	−0.5338	1.4713	1.5407	0.2504	0.5152	−1.5131	−0.2895	0.5733	8,039,038.2128	266,875.1745	135.62343713	4.50234561	−2,613.0316
WTS Ground Location	−2.3290	−0.5224	1.8509	1.5539	0.2391	0.8948	−1.5084	−0.2976	0.9532	8,039,038.2047	266,875.1792	135.62343699	4.50234569	−2,613.4115
HP3 Original Hook	−1.8881	0.5541	1.3900	1.1130	−0.8374	0.4339	−1.0965	0.8047	0.5278	8,039,039.3070	266,875.5911	135.62345559	4.50235264	−2,612.9861
HP3 Original Ground Location	−1.8960	0.5800	1.8392	1.1209	−0.8634	0.8831	−1.0833	0.8078	0.9776	8,039,039.3101	266,875.6043	135.62345564	4.50235287	−2,613.4359
HP3 Mole Cap Sol 407	−1.9458	0.6237	1.7812	1.1707	−0.9070	0.8252	−1.1361	0.8538	0.9244	8,039,039.3562	266,875.5515	135.62345642	4.50235198	−2,613.3827
RISE +*Y*	−0.1636	0.6057	0.8730	−0.6114	−0.8891	−0.0830	0.6013	0.8974	−0.0660	8,039,039.3998	266,877.2889	135.62345715	4.50238129	−2,612.3923
RISE −*Y*	−0.0137	−0.6028	0.8730	−0.7614	0.3195	−0.0830	0.7586	−0.3083	−0.1348	8,039,038.1941	266,877.4462	135.62343681	4.50238394	−2,612.3235
Magnetometer	−0.5861	0.6745	0.9955	−0.1890	−0.9578	0.0394	0.1846	0.9562	0.0794	8,039,039.4585	266,876.8722	135.62345814	4.50237426	−2,612.5377
Transducer	0.1541	0.3626	1.1090	−0.9292	−0.6460	0.1530	0.9313	0.6454	0.1421	8,039,039.1477	266,877.6189	135.62345290	4.50238685	−2,612.6004
Pressure Port	0.2190	0.1763	0.9536	−0.9941	−0.4597	−0.0024	0.9900	0.4678	−0.0255	8,039,038.9701	266,877.6775	135.62344990	4.50238784	−2,612.4328
TWINS +*Y* Base Mast	−0.0697	0.5574	0.9564	−0.7054	−0.8407	0.0003	0.6993	0.8457	0.0103	8,039,039.3480	266,877.3869	135.62345628	4.50238294	−2,612.4686
TWINS +*Y* Temperature Sensor	−0.0330	0.6334	0.7627	−0.7421	−0.9167	−0.1934	0.7264	0.9317	−0.1808	8,039,039.4341	266,877.4140	135.62345773	4.50238340	−2,612.2775
TWINS +*Y* Wind Sensor	−0.0329	0.6901	0.7021	−0.7422	−0.9734	−0.2540	0.7233	0.9914	−0.2384	8,039,039.4938	266,877.4108	135.62345874	4.50238334	−2,612.2199
TWINS −*Y* Base Mast	−0.1401	−0.5386	0.9560	−0.6350	0.2553	−0.0001	0.6358	−0.2495	−0.0429	8,039,038.2529	266,877.3234	135.62343780	4.50238187	−2,612.4154
TWINS −*Y* Temperature Sensor	−0.1769	−0.6147	0.7623	−0.5982	0.3313	−0.1938	0.5905	−0.3159	−0.2383	8,039,038.1865	266,877.2780	135.62343668	4.50238110	−2,612.2200
TWINS −*Y* Wind Sensor	−0.1769	−0.6713	0.7017	−0.5982	0.3880	−0.2544	0.5879	−0.3694	−0.3016	8,039,038.1330	266,877.2755	135.62343578	4.50238106	−2,612.1567

### 3.5 Magnetometer Location

**Figure 10:**
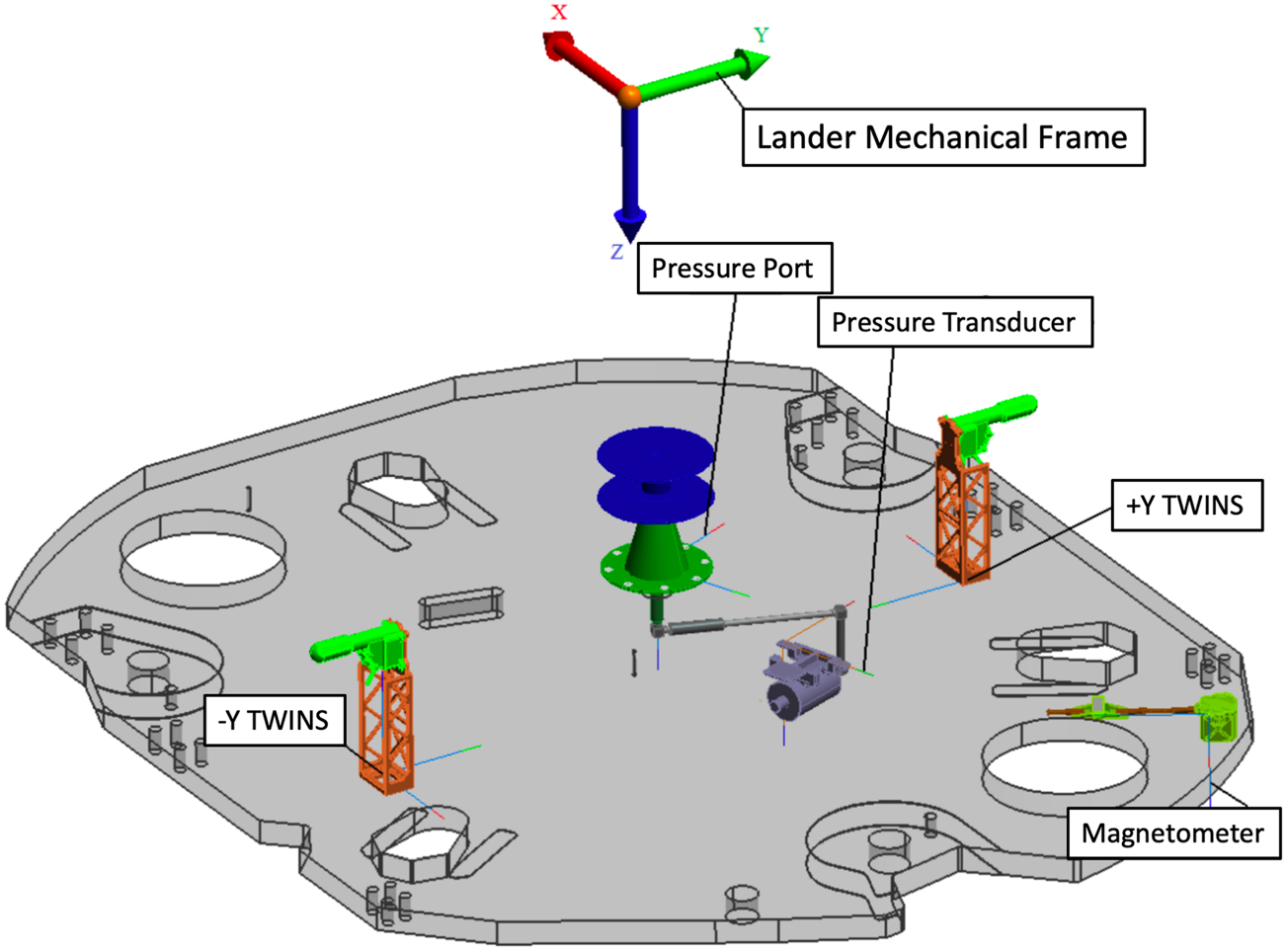
Diagram of the lander deck from above showing the pressure sensor and port, the magnetometer, and the −*Y* and +*Y* TWINS masts in the lander mechanical frame. The lander mechanical frame origin shown (color arrows) is about 1 m above the lander deck. The location of the RISE antennas are near the TWINS booms (see Figures 8 and 12).

The magnetometer is located on the underside of the lander, near the −*X*, +*Y* edge of the deck in the spacecraft mechanical frame (Figure 10), with its coordinate frame axes measured relative to the spacecraft mechanical frame as:

(8) $${\left[\begin{array}{ccc} x_{x\text{axis}} & x_{y\text{axis}} & x_{z\text{axis}}\\ y_{x\text{axis}} & y_{y\text{axis}} & y_{z\text{axis}}\\ z_{x\text{axis}} & z_{y\text{axis}} & z_{z\text{axis}} \end{array}\right]}_{\mathrm{SM}}=\left[\begin{array}{ccc} -0.848561 & 0.529097 & -0.000117\\ -0.529097 & -0.848561 & -0.000523\\ -0.000376 & -0.000382 & 1.000000 \end{array}\right]$$

To rotate the three axes into the cartographic frame (northing/easting/elevation‐up in meters), each axis vector is rotated by the SM to IDA to site frame rotations (ignoring the SM to IDA origin translation) as well as a mirroring about the *XY* axis to flip the vertical component to the opposite of gravity:

(9) $$\left[\begin{array}{ccc} Northing_{x\text{axis}} & Northing_{y\text{axis}} & Northing_{z\text{axis}}\\ Easting_{x\text{axis}} & Easting_{y\text{axis}} & Easting_{z\text{axis}}\\ {\mathrm{Up}}_{x\text{axis}} & {\mathrm{Up}}_{y\text{axis}} & {\mathrm{Up}}_{z\text{axis}} \end{array}\right]=\begin{array}{c} \underset{\text{Flip Vertical}}{\underbrace{\left[\begin{array}{ccc} 1 & 0 & 0\\ 0 & 1 & 0\\ 0 & 0 & -1 \end{array}\right]}} \end{array}*\begin{array}{c} \underset{\mathrm{IDA}\;\text{to Site}}{\underbrace{\left[\begin{array}{ccc} -0.99887 & 0.00622310 & 0.047204\\ -0.0086214 & -0.99867 & -0.050774\\ 0.046825 & -0.051123 & 0.99759 \end{array}\right]}} \end{array}*\begin{array}{c} \underset{\mathrm{SM}\;\text{to}\;\mathrm{IDA}}{\underbrace{\left[\begin{array}{ccc} -1 & 0 & 0\\ 0 & -1 & 0\\ 0 & 0 & 1 \end{array}\right]}} \end{array}*\underset{\mathrm{Mag}\;\text{Axes in}\;\mathrm{SM}}{\underbrace{{\left[\begin{array}{ccc} x_{x\text{axis}} & x_{y\text{axis}} & x_{z\text{axis}}\\ y_{x\text{axis}} & y_{y\text{axis}} & y_{z\text{axis}}\\ z_{x\text{axis}} & z_{y\text{axis}} & z_{z\text{axis}} \end{array}\right]}_{\mathrm{SM}}}}$$

The resulting axes are oriented in the cartographic frame as follows.

**Table:** 

	*X* Axis	*Y* Axis	*Z* Axis
Northing Component	−0.8443	−0.5357	−0.0123
Easting Component	0.5338	−0.8429	0.0685
Elevation‐Up Component	0.0471	−0.0513	−0.9976

The *XYZ* location of the magnetometer provided in spacecraft mechanical frame is −23.076, 26.554, and 39.192 in, yielding the location and elevation of the center of the magnetometer shown in Table 2. At this location, the long axis of the magnetometer (*Y* axis) is oriented roughly northwest, with the azimuth from north calculated as arctan(0.8429/0.5357) = 57.56° measured counter‐clockwise from north, or 302.44° measured clockwise from north. The *X* axis of the magnetometer is orthogonal to the *Y* axis, toward the southwest with an azimuth of 212.44° measured clockwise from north.

### 3.6 Meteorology Instrument Locations

The atmospheric pressure sensor (transducer) is located inside the spacecraft with an inlet pressure port near the center of the lander deck (Figure 10). The Transducer *XYZ* provided in spacecraft mechanical frame is as follows: 6.067, 14.276, and 43.663 in, yielding a location shown in Table 2. The inlet Pressure Port *XYZ* provided in spacecraft mechanical frame is as follows: 8.622, 6.942, and 37.544 in, yielding a location on Mars shown in Table 2.

**Figure 11:**
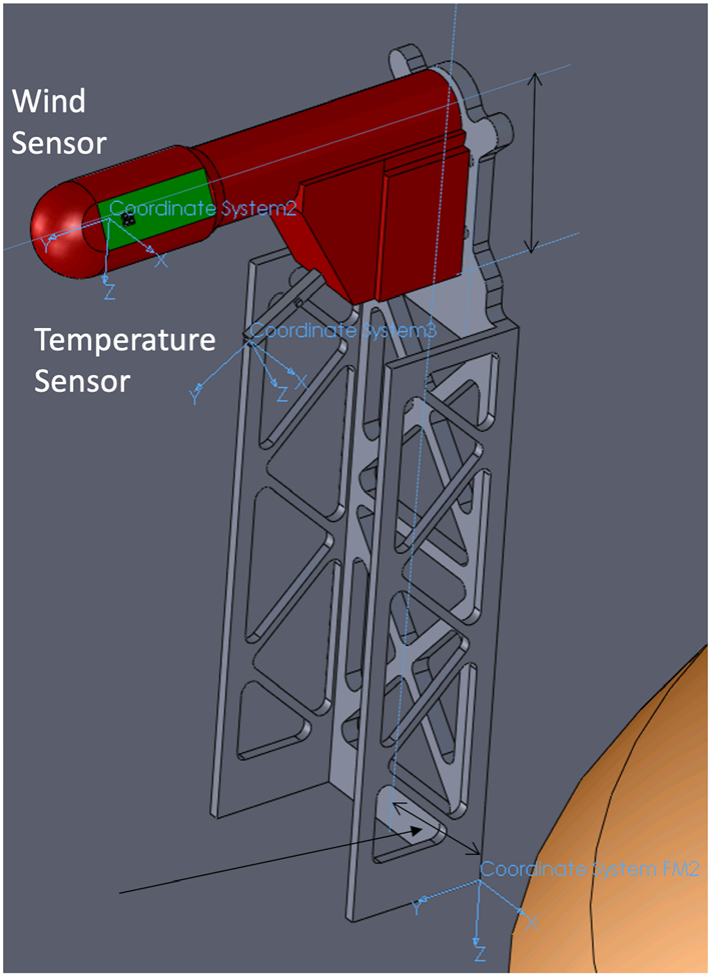
Diagram of a TWINS mast showing the location of the base corner of the mast on the deck, which is the location in the SM frame, and the temperature and winds sensors. The wind sensor is about 25 cm above the lander deck.

The Temperature and Winds for InSight (TWINS) masts are located at the edge of the lander deck in the +*Y* and −*Y* directions in the spacecraft mechanical frame (Figure 10). The base corner of the masts on the lander deck are reported in Table 2. The TWINS temperature and wind sensors are located atop the masts and offset from the coordinates of the base of the masts as shown in the Figure 11. Measured in the SM frame, the thermometer is offset by (−36.75, 76.02, −193.69) mm from the base corner of the mast, and the wind sensor is offset by (−36.75, 132.70, −254.28) mm. Applying the appropriate orientation rotations for the +*Y* (−1; 1; 1) and −*Y* (1; −1; 1), these offsets are added to the locations of the TWINS bases in the SM frame to obtain their locations on Mars (Table 2).

### 3.7 RISE Antenna Locations

Two medium gain antennas are mounted on the top deck in the +*Y* and −*Y* direction in the SM frame and are used for X‐band direct to Earth radio tracking by RISE. Both antennas point 30° above the deck with one (+*Y*) pointed 15.5° clockwise from the +*Y* direction and the other (−*Y*) pointed 186° clockwise from the +*Y* direction in the SM frame (Folkner et al., 2018). The base of the cone shaped antennas is attached to support structures along a circular mounting interface whose diameter is 3 in. The XYZ of the lowest point on the mounting circle in the SM frame is −6.4424, 23.8483, and 34.3711 in for the +*Y* antenna and −0.5385, −23.7336, and 34.3711 in for the −*Y* antenna. Applying the transformations to IDA and site frames yields the cartographic locations for the two antennas shown in Table 2.

### 3.8 Coordinates Summary

**Figure 12:**
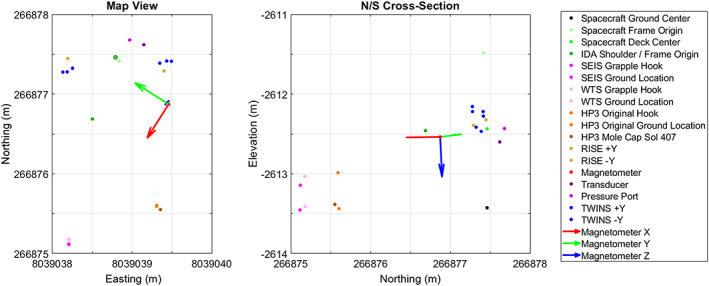
Summary of the locations of the instruments in cartographic space on Mars. (a) The location in northing and easting and (b) northing and elevation. Three points for the TWINS booms are the base, temperature, and wind sensors shown in Figure 11. Black outline of spacecraft deck center is to show that the spacecraft ground center is directly beneath this point.

The locations of the spacecraft and instruments in cartographic space are summarized in Figure 12. The map view shows the locations in northing and easting. The farthest north instrument is the pressure sensor and inlet port. The farthest south instrument is the SEIS (and WTS). The farthest east instrument is the +Y TWINS mast and temperature and wind sensors, and the farthest west point is −*Y* TWINS mast and temperature and wind sensors, with the magnetometer close by. The north‐south extent of the instruments is ~2.5 m and the east‐west extent is a little over 1 m. The N‐S cross section shows the SEIS ground location is the lowest elevation and the +*Y* TWINS boom is the highest elevation. The distance between SEIS and HP^3^ is 1.227 m measured horizontally in site frame. SEIS is located 1.595 m from the lander measured horizontally in site frame from the IDA shoulder joint. The HP3 mole is located 1.421 m from the lander measured horizontally in site frame from the IDA shoulder joint.

## 4 Setting, Viewshed, and Nearby Features

In this section of the paper, we explore the setting of the InSight lander on the surface in terms of topography, nearby features, and what can be seen in the panoramas. This is based on the lander images, the orthoimages, and the DEM (and is less quantitative than previous material). A viewshed map of areas within view from a height of 1.5 m at the lander location (the average height of the IDC when acquiring panorama images) was made using the HiRISE DEM of the landing site (InSightE17_C; Fergason et al., 2017) and finding clear lines of sight from this location to the first obstruction at all azimuths.

As all other surface missions have done, prominent surface features in the panoramas have been given names for communications purposes. These are all informal names and are not official IAU names. Almost all of the names were proposed by members of the InSight geology theme group or the ISSWG. Also, as done for previous missions, no personal, political, offensive names, or names of people were allowed (M. Golombek and N. Williams served as the name czars). Names were assigned in IDC panoramas for features in the middle and far field, in ISSWG mosaics of features in the workspace, and in ICC and IDC images for areas near the lander.

**Figure 13:**
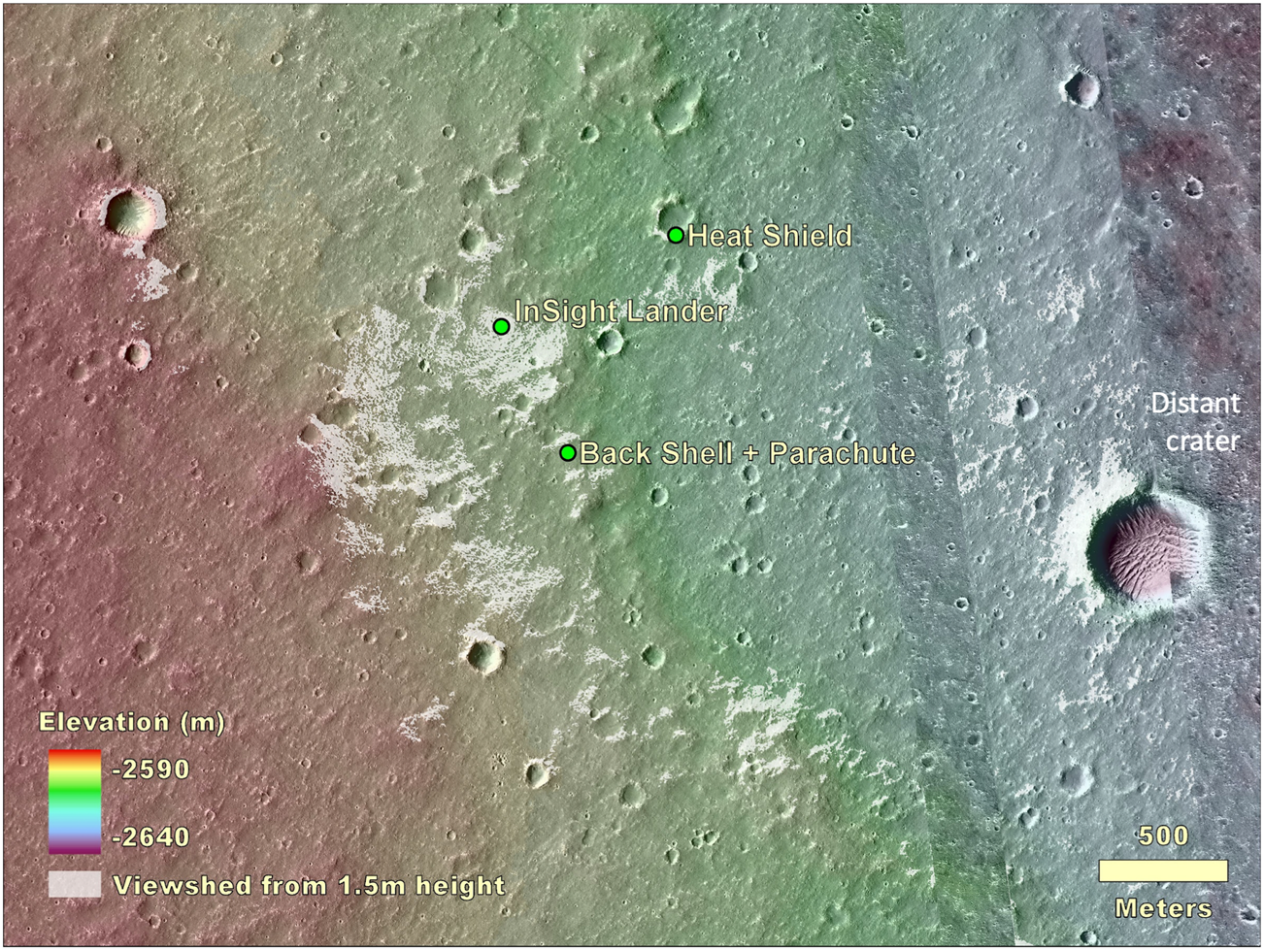
Combined topographic and viewshed map overlain on a HiRISE image of the area within several kilometers of the InSight lander, heatshield, and back shell/parachute. The viewshed at a height of 1.5 m above the lander location is shown in white. Distant crater, 2.4 km to the southeast, is farthest feature observed from the spacecraft. The topographic map is from a HiRISE stereo pair produced by Fergason et al. (2017) (InSightE17_C) produced during the landing site selection effort (Golombek et al., 2017).

**Figure 14:**
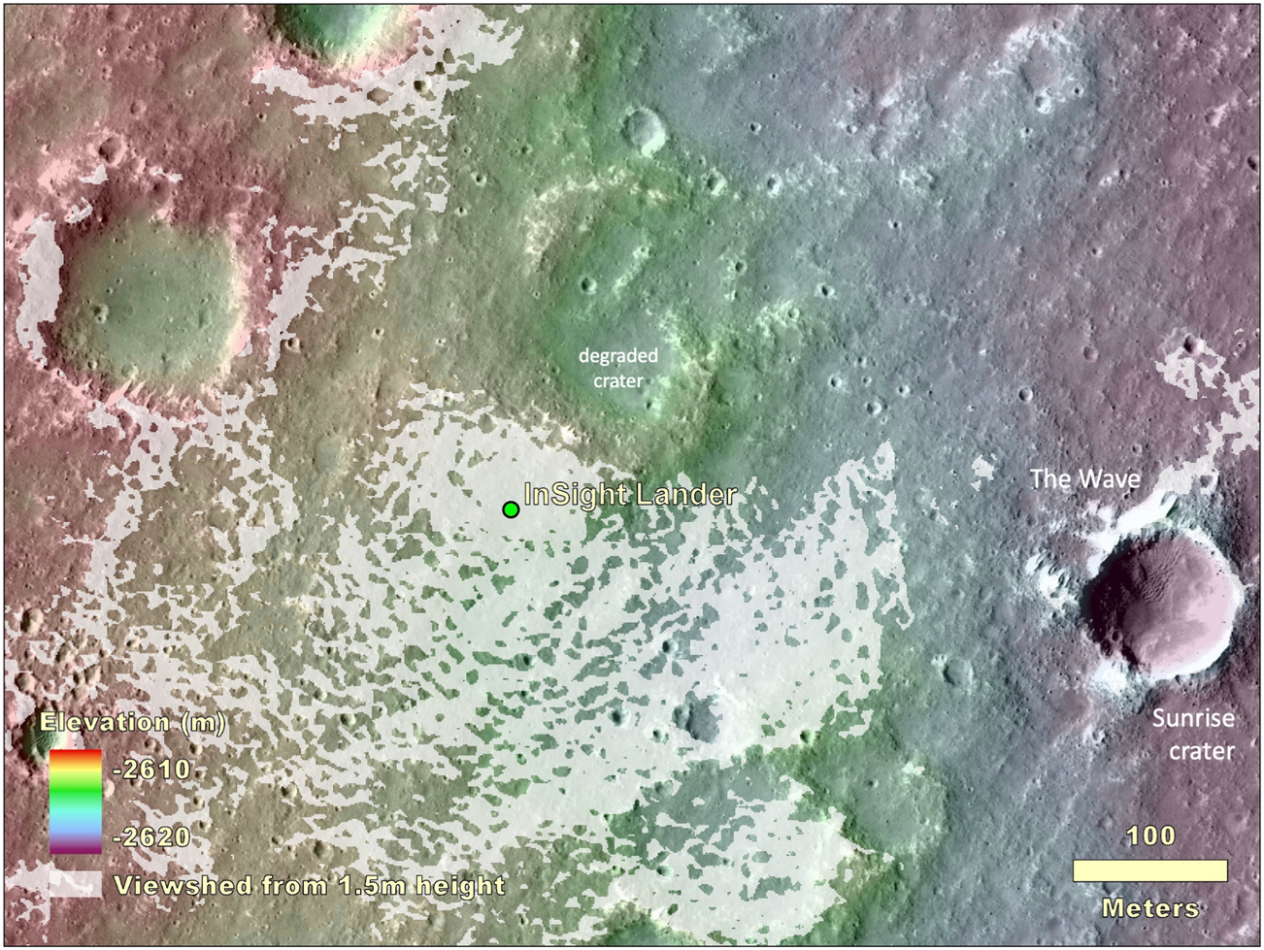
Combined topographic and viewshed map overlain on a HiRISE image of the area within hundreds of meters of the InSight lander. The viewshed at a height of 1.5 m above the lander location is shown in white. Note Sunrise crater and eolian bedforms, The Wave, 400 m to the east. The topographic map is from a HiRISE stereo pair produced by Fergason et al. (2017) (InSightE17_C) produced during the landing site selection effort (Golombek et al., 2017).

**Figure 15:**
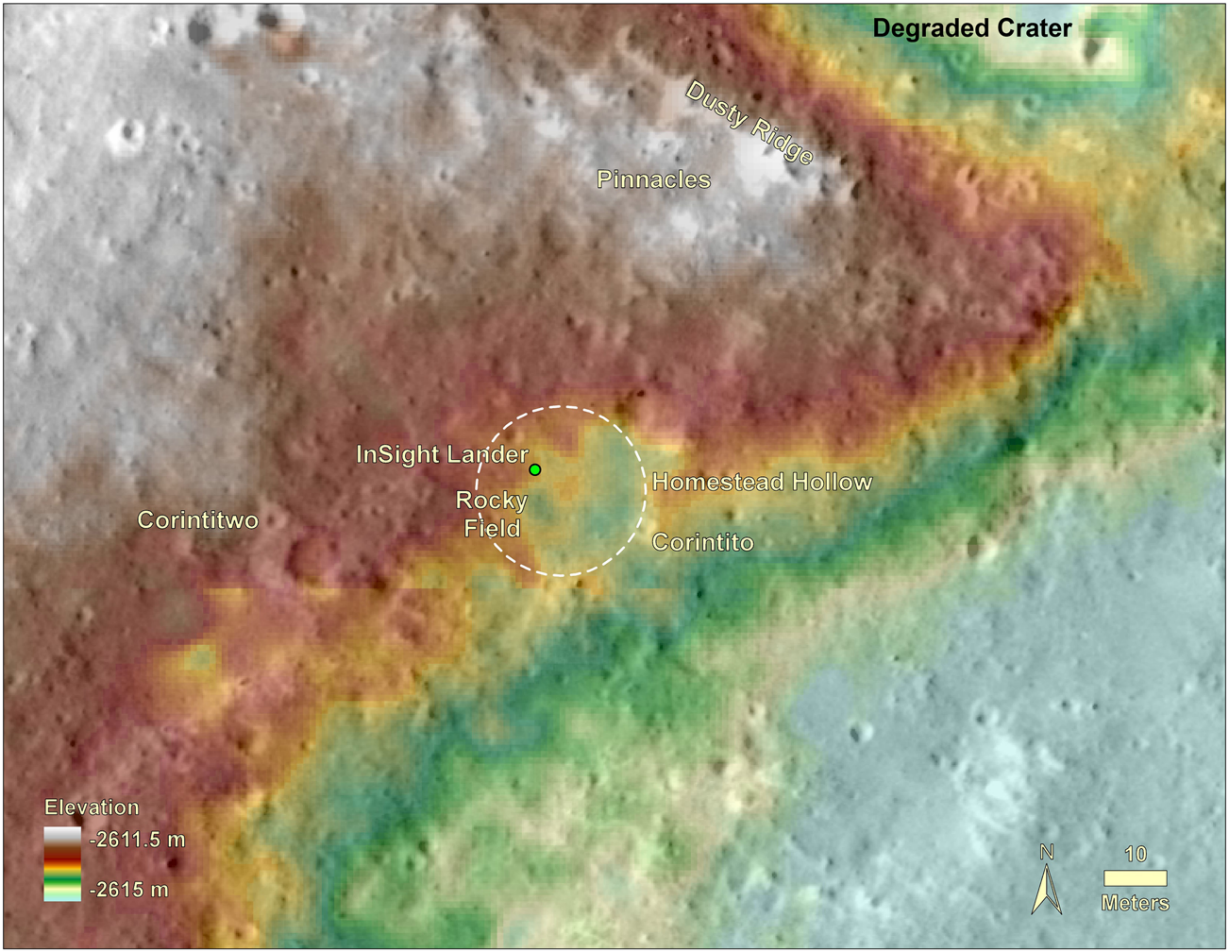
Topographic map overlain on a HiRISE image of the area around InSight. The lander is located in the northwest portion of a 27 m diameter quasi‐circular depression, Homestead hollow (dashed circle). The hollow is around 0.3 m deep on average (varies from 0.2 to 0.5 m) with higher topography around it. Two Corinto secondary craters (Corintito and Corintitwo) are in view of the lander as are three rocks (The Pinnacles) and an eolian bedform (Dusty Ridge) to the north‐northeast. Note craters in widely varying states of degradation from very fresh (Corinto secondaries) to highly degraded Homestead hollow. Digital elevation model (color) from stereo HiRISE images from Fergason et al. (2017) (InSightE17_C).

The lander is located on the western side of a quasi‐circular depression that has been interpreted to be a degraded ~27 m diameter impact crater (Warner et al., 2020), informally named Homestead hollow (Figures 13, 14, 15). Homestead hollow to the east is a smooth, sandy, and granule‐ and pebble‐rich surface. To the west is slightly rockier and rougher terrain (Golombek, Warner, et al., 2020). Craters in a wide variety of degradational states are common in the high‐resolution orbital images. A slope up to the north limits the horizon to about 50 m away; it is topped by three rocks (The Pinnacles), and eolian bedforms (Dusty ridge) near the southwest rim of a ~100 m diameter degraded impact crater (Figure 15). To the east‐southeast (Figure 14), the horizon extends about 400 m to the rim of a relatively fresh, ~100 m diameter impact crater (Sunrise) with large eolian bedforms on its rim (The Wave). The rim of a larger (460 m diameter), relatively fresh crater can be seen on the east‐southeast horizon, ~2.4 km away (Distant crater in Figure 13) (Golombek, Warner, et al., 2020).

At the several kilometer scale, the viewshed map shows the lander is on a broad gentle slope down to the east (Figure 13). The total relief is around 50 m over a distance of 4.5 km, yielding a slope of less than 1° (0.6°). This gentle slope reveals a crater rim over 2 km away to the east in contrast to the west, where the viewshed extends only about 500 m (Figure 13). The DEM and viewshed indicates the backshell and parachute are in a slight depression and should not be visible from the lander. The heatshield is in an area that should be in view, but it has not been identified in the lander images. A likely factor is the resolution of the IDC where the pixel scale at 762 m distance (to the heatshield) is only ~0.6 m, so the maximum possible height of the heatshield if it were on its side (cone shape) would span at most only ~1 pixel.

The farthest feature observed from the lander is the rim of Distant crater 2.4 km to the east‐southeast (Figure 13). Distant crater is a relatively fresh ~460 m diameter simple, rocky ejecta crater. A relatively fresh crater with this morphology (class 2; Sweeney et al., 2018) in the landing ellipse has a rim height that is 0.029 times its diameter, so its rim is about 14.5 m high, which easily stands above the surroundings. The relatively fresh, 250 m diameter rocky ejecta crater about 1.5 km to the west‐northwest of the lander should be visible according to the viewshed, but is indistinct in IDC images.

On the scale of several hundred meters, the lander is on a shallow slope (0.7°) down to the east (Figure 14). The viewshed is fairly continuous out to about 50 m. Beyond that distance, the most continuous views are to the southeast. To the east‐southeast (Figure 14), the horizon extends around 400 m to the rim of Sunrise crater. To the northeast (Figure 14), a 2.2° slope limits the horizon to about 50 m to The Pinnacles and Dusty ridge.

We have identified the azimuths (measured clockwise from north) of features that are in view in both the surface panoramas and the HiRISE image. If the yaw, pitch, and roll of spacecraft and the transformation from the spacecraft mechanical frame to the site frame are known perfectly and there are no errors in georeferencing of the HiRISE orthophoto, then the azimuths measured in the panoramas and on the HiRISE orthophoto of the same features should match. Table 3 shows all craters, rocks, and other features that have been identified in both the lander panoramas and in HiRISE. The azimuths of almost all features matches to within 1°. The best matched features greater than 50 m away (Pinnacle rocks, Dusty ridge, The Wave, and Distant crater) agree to an average of 0.5°. This suggests that the orientation (including the azimuth and tilt) recorded by the IMU (Inertial Measurement Unit) and used to determine the site frame are accurate to better than 1°.

**Table 3:** Craters, Rocks, and Eolian Bedforms Identified in Both Lander Panoramas and HiRISE

Feature name	Azimuth in IDC (deg)	Azimuth in HiRISE (deg)	Distance in HiRISE (m)	Crater Diam (m)
Pinnacle W Rock	21	22	56.0	
Pinnacle C Rock	24	24	54.8	
Pinnacle E Rock	28	28	60.0	
Dusty Ridge West Peak	35	35	62.7	
Slippery Rock	39	40	13.8	
Hanging Rock	52	53	20.7	
Knee Deep Crater	61	61	18.2	7
The Wave North Peak	90	91	422.5	
Distant Crater's North Rim	104	105	2534.0	
Table Rock	104	106	17.6	
School House Rock	105	107	19.6	
Corintito Crater	116	118	19.8	3.1
Cone Rock	127	128	32.8	
Flat Top Rock	136	138	14.8	
Puddle Crater	144	145	9.6	1.5
Peekaboo Crater	158	158	20.6	3.5
First Rock	160	160	19.4	
Kettle Crater	161	161	15.5	1.7
Mailbox Rock	212	212	22.2	
Deep Cut Crater	224	221	10.2	1.8
Squash Crater	250	247	38.8	8.8
Corintitwo Crater	256	259	42.3	3
Coffee Crater	266	263	6.2	0.87
Smudge Crater	278	278	14.9	2.8
Sunset Crater	284	282	25.0	10
Churro Rock	299	299	22.8	
Campfire Crater	314	313	19.8	1.7
Blast Zone Crater	329	329	12.5	1.2
Blast Zone Hollow	332	332	12.8	3.2
Near Miss Crater	343	326	6.2	2.9
Mole Crater	344	343	16.6	4
Hedgehog Rock	346	347	21.5	
Gazebo Rock	347	347	35.4	
Slug Rock	353	354	21.6	

*Note*. Azimuth measured clockwise from north.

## 5 Description of Panoramas and Features in View

**Figure 16:**
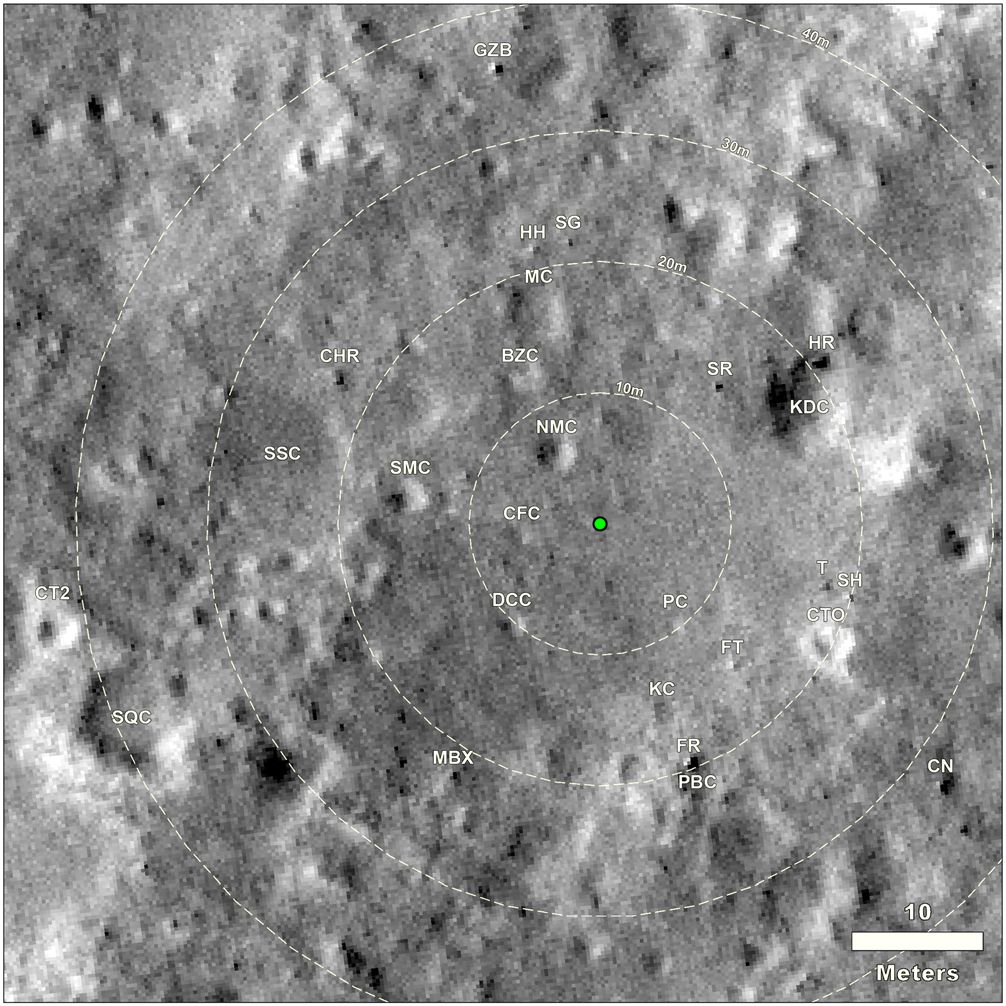
HiRISE image of the area around the InSight lander (green dot) with craters and rocks that can be identified in both the panoramas (next figures) as well as this orbital image. Clockwise from due north (up on the image), abbreviations are as follows: SR is Slippery rock, HR is Hanging rock, KDC is Knee Deep crater, T is Table rock, SH is School House rock, CTO is Corintito crater, CN is Cone rock, FT is Flat Top rock, PC is Puddle crater, PBC is Peekaboo crater, FR is First rock, KC is Kettle crater, MBX is Mail Box rock, DCC is Deep Cut crater, SQC is Squash crater, CT2 is Corintitwo crater, CFC is Coffee crater, SMC is Smudge crater, SSC is Sunset crater, CHR is Churro rock, CPF is Campfire crater, BZC&H is Blast Zone crater and hollow, NMC is Near Miss crater, MC is Mole crater, HH is Hedgehog rock, GZB is Gazebo rock, and SG is Slug rock. HiRISE image number ESP_036761_1845, not map projected, that has been georeferenced into a map view.

We will describe features within the IDC panorama, split into eighths (45° with ±5° overlap). We use portions of the evening and afternoon panoramas to highlight features better resolved in those lighting conditions, which are controlled stereo panoramas projected into site frame. We will then describe features in the workspace and area near the lander. We also include an annotated HiRISE image with many of the same features shown (Figure 16). The distance and azimuth of the craters and rocks within view and identified in HiRISE as well as crater diameters are included in Table 3.

**Figure 17:**
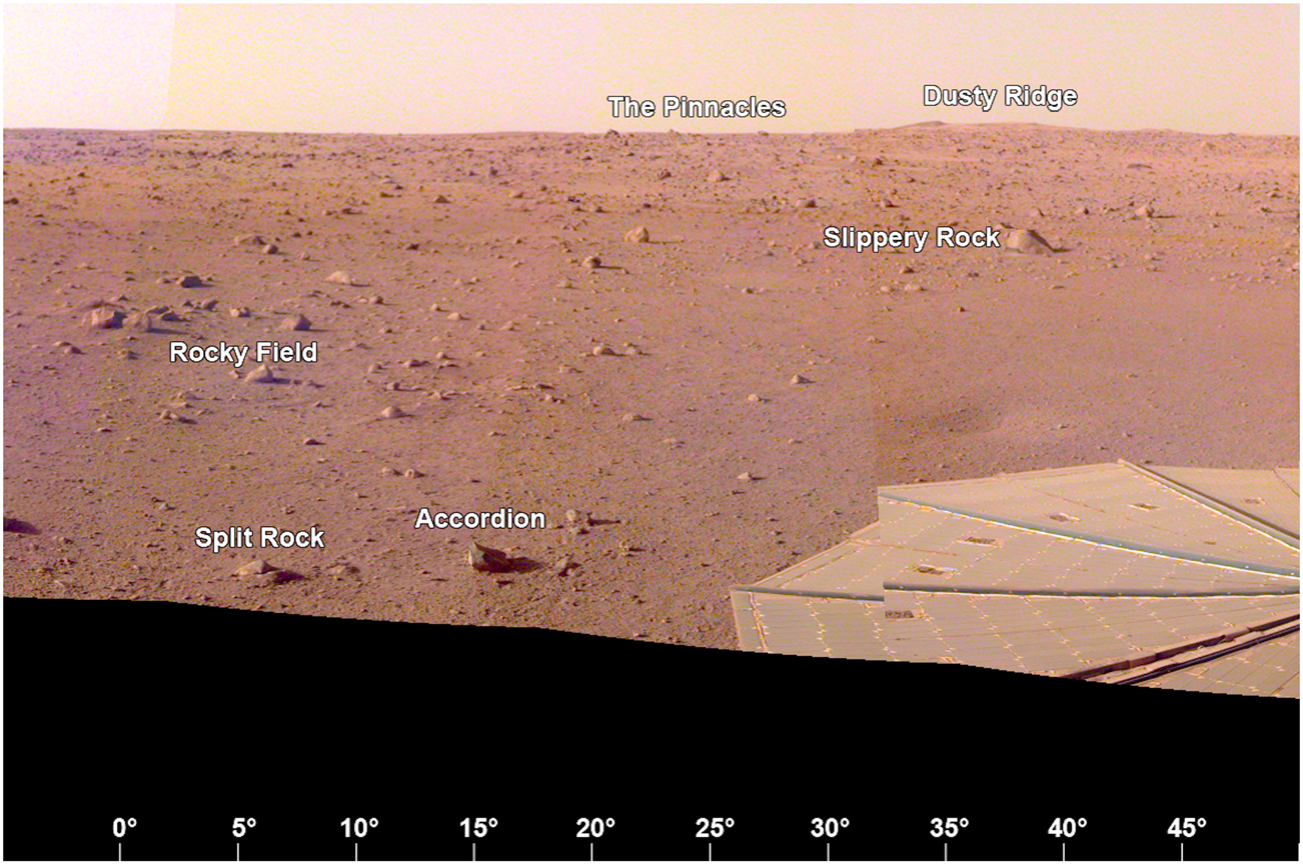
The view from the lander looking to the north‐northeast (0° to 50°) showing part of the +*Y* SM frame solar panel, Rocky field, the smooth terrain to the east (right) and rockier terrain outside Homestead hollow. On the horizon are The Pinnacles rocks (three) and Dusty ridge, an eolian bedform, which are near the rim of a ~100 m diameter degraded impact crater about 50 m away. A portion of the evening panorama that has been stretched and is not true color.

The area to the north‐northeast of the lander (0–45° azimuth, clockwise from north, Figure 17) is characterized by the contact between Rocky Field and the smooth terrain of Homestead hollow at an azimuth of 30° (i.e., north 30° east). Rocky Field is the rocky portion of Homestead hollow to the west of the lander. On the horizon to the north‐northeast are The Pinnacles rocks (three) and Dusty ridge, an eolian bedform, which are near the rim of a ~100 m diameter degraded impact crater about 50 m away. The rim of Homestead hollow is around ~20 m away, beyond which the terrain is slightly brighter (dustier) and rockier. There are about 10 rocks in Rocky Field that are ~5–10 cm diameter. Most of them appear perched and many appear angular. They have been interpreted as being deposited by a nearby impact after Homestead hollow was filled in (Grant et al., 2020; Warner et al., 2020) The largest rock near the rim of Homestead hollow in this view is Slippery rock ~14 m from the lander.

**Figure 18:**
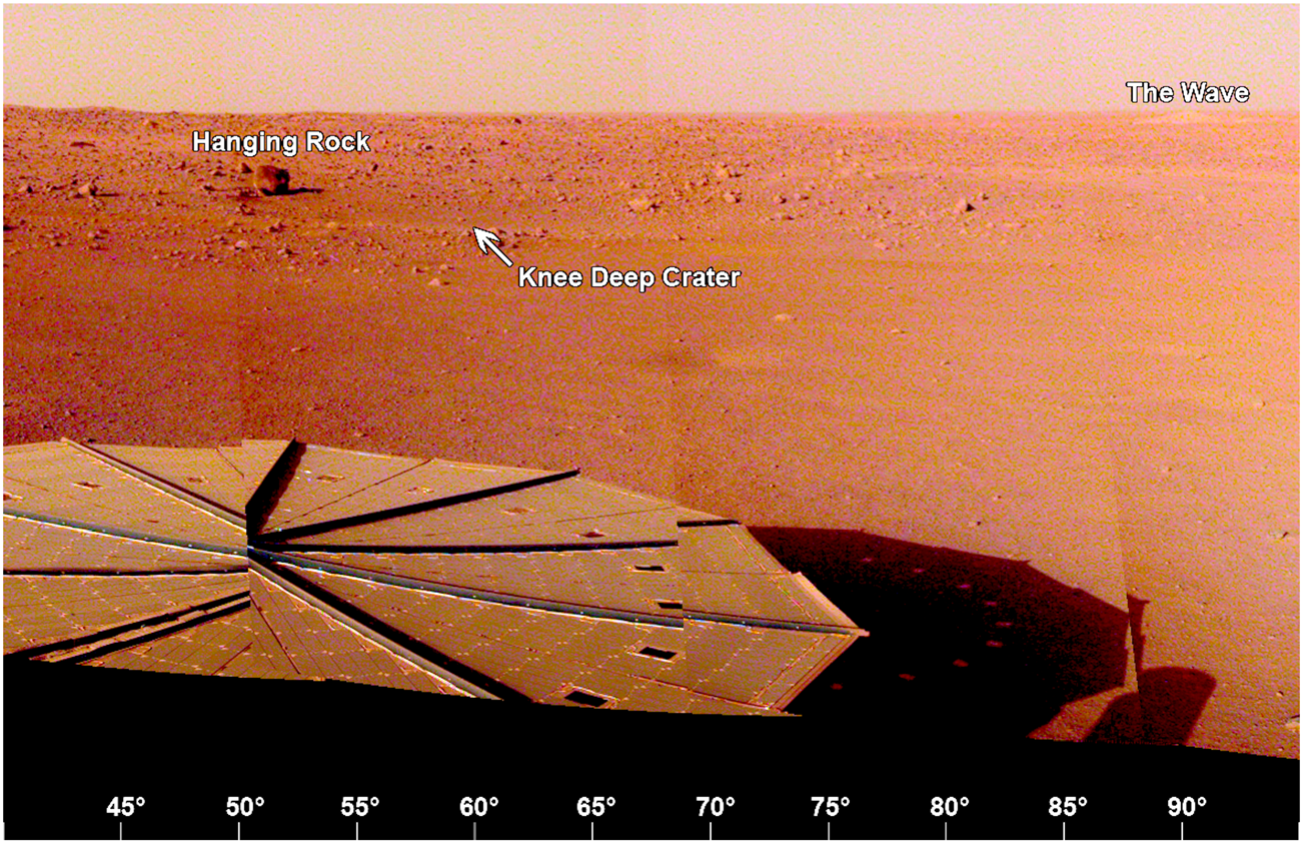
The view from the lander looking to the east‐northeast (40° to 95°) showing +*Y* SM frame solar panel, the smooth terrain of Homestead hollow out to around 15 m, with Knee Deep crater and the tabular Hanging rock beyond. The eolian bedform, The Wave is on the rim of a relatively young, 100 m diameter rocky ejecta crater (Sunrise) that is around 400 m away to the east. A portion of the evening panorama that has been stretched and is not true color.

The area to the east‐northeast (45° to 90° azimuth, Figure 18) from the lander is characterized by smooth flat Homestead hollow terrain with no large rocks within around 15 m of the lander. The solar panel obscures the area nearest the lander. Near the rim of Homestead hollow is Knee Deep crater (60°), a shallow sandy depression, and Hanging rock, a perched rock, around 21 m away. The horizon away from Dusty Ridge and the eroded crater is fairly flat. A bright eolian bedform, The Wave, is near the horizon at an azimuth of ~90°.

**Figure 19:**
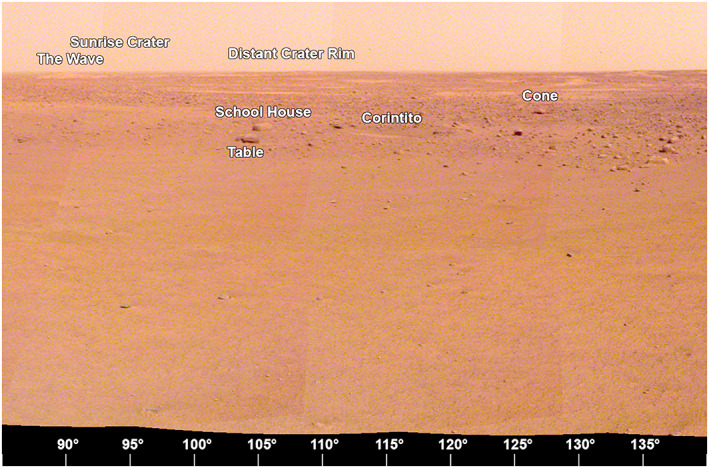
The view from the lander looking to the east‐southeast (90° to 140°) showing the smooth terrain of Homestead hollow out to around 15 m, with Table, Schoolhouse and Cone rocks and Corintito crater. In the distance, The Wave, a bright eolian bedform and the Sunrise crater rim, a relatively young, 100 m diameter rocky ejecta crater are around 400 m away at an azimuth of 90°. The rim of a larger (460 m diameter), relatively fresh Distant crater can be seen on the east‐southeast horizon (~107° azimuth) ~2.4 km away. A portion of the afternoon panorama that has been stretched and is not true color.

The area to the east‐southeast within 15 m of the lander (90° to 135° azimuth, Figure 19), is composed of smooth terrain of Homestead hollow with very few rocks. Two relatively large, tabular, perched rocks, School House and Table rocks are about 18 and 20 m away, respectively, at an azimuth of 105°. A secondary crater that appears to be from Corinto crater (IAU name), called Corintito crater is ~19 m away. It is 2.95 m in diameter and has the bright ejecta in HiRISE images characteristic of Corinto secondary craters that are ubiquitous across the landing ellipse (Golombek et al., 2017; Golombek, Kass, et al., 2020). Corinto crater is located ~800 km to the north‐northeast of the landing site. It has a diameter of 13.9 km and is one of the youngest bright rayed craters on Mars (McEwen et al., 2005; Preblich et al., 2007; Tornabene et al., 2006). It spewed dense swarms of secondary craters 1,500–2,000 km to the south. Mapping of the relative age of Corinto and Zunil (IAU names) secondaries on terrains indicates Corinto impacted before Zunil (0.1–1 Ma) but after a young 2.5 ± 0.2 Ma Elysium lava flow (Bloom et al., 2014; Golombek et al., 2014; Hundal et al., 2017). Corintito has a sandy, shallow parabolic floor with a low depth/diameter (0.05) ratio, which is consistent with general expectations for Corinto secondaries based on prelanding orbital images (Golombek et al., 2017; Golombek, Kass, et al., 2020). In the distance, The Wave, a bright eolian bedform and the rim of Sunrise crater, a relatively young, 100 m diameter rocky ejecta crater are both ~400 m away at an azimuth of 90° (due east). The rim of a larger (460 m diameter), relatively fresh crater can be seen on the east‐southeast horizon (107° azimuth), ~2.4 km away (Distant crater in Figure 13). This is the most distant feature observed from the lander that can also be identified in HiRISE. There are also a large number of unnamed, very shallow, bright quasi‐circular depressions interpreted to be degraded and filled impact craters (hollows) visible between the lander and the horizon.

**Figure 20:**
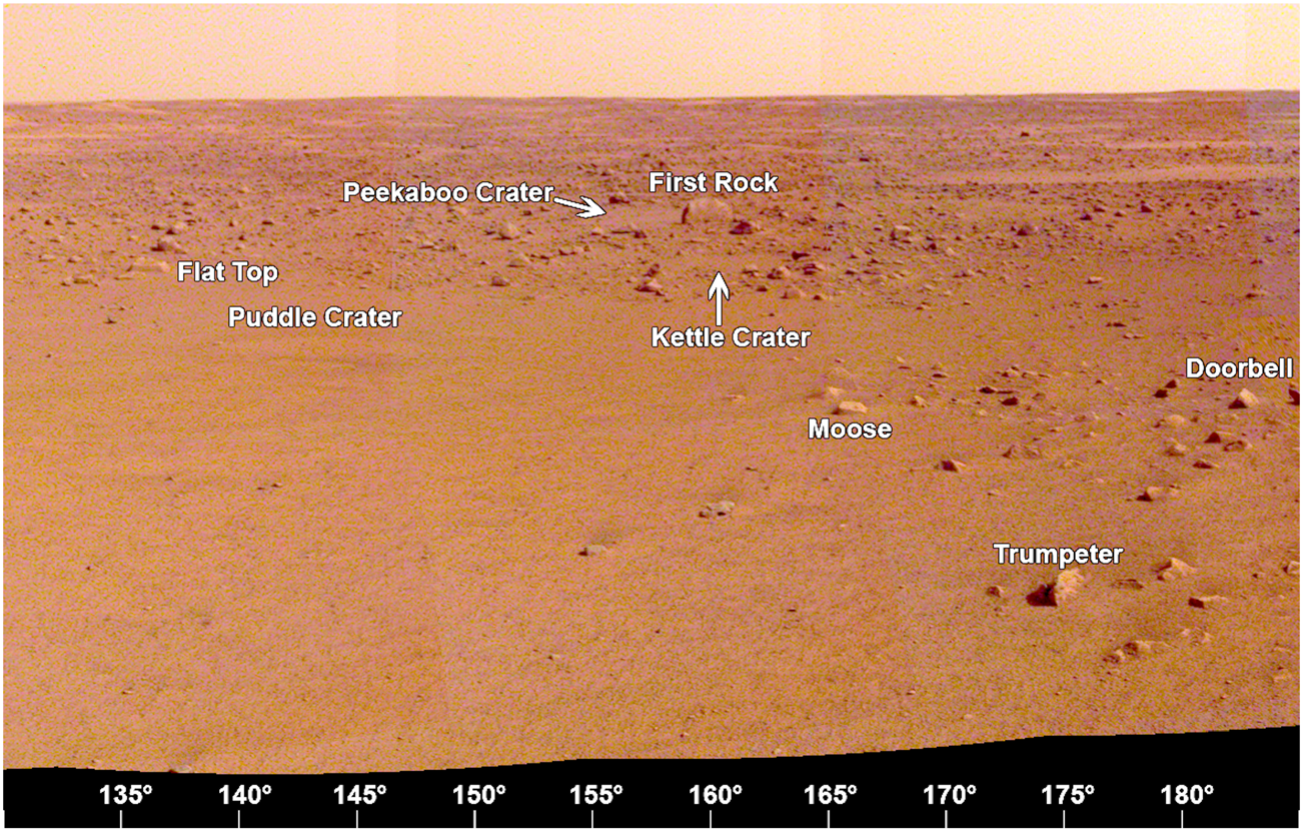
The view from the lander looking to the south‐southeast (130° to 185°) showing the contact between the smooth terrain of Homestead hollow with the rockier terrain to the west. Several small craters (Puddle and Kettle) and rocks can be seen, including First rock, so named because it was the first large rock seen from the IDC before the panoramas were acquired. A portion of the afternoon panorama that has been stretched and is not true color.

The area to the south‐southeast (135–180° azimuth, Figure 20) includes the contact (at an azimuth of 170°) between smooth, relatively rock free terrain to the east and rockier terrain to the west. The rim of Homestead hollow is ~15 m away with several small craters (Puddle, Kettle, and Peekaboo) just beyond. All three craters are shallow sandy depressions. The largest rock in view, called First Rock, because it was the first large rock identified from the lander in one of the initial IDC images of the surface around the lander, is ~20 m away at an azimuth of 160°. It is adjacent to Peekaboo crater, and it is tabular and appears perched, similar to many of the smaller rocks around it. A large number of small rocks litter the floor of Homestead hollow, including Moose at 165° and Trumpeter at 175°. The terrain beyond the hollow includes many bright hollows and bright, sandy regions that extend to the horizon that is hundreds of meters away.

**Figure 21:**
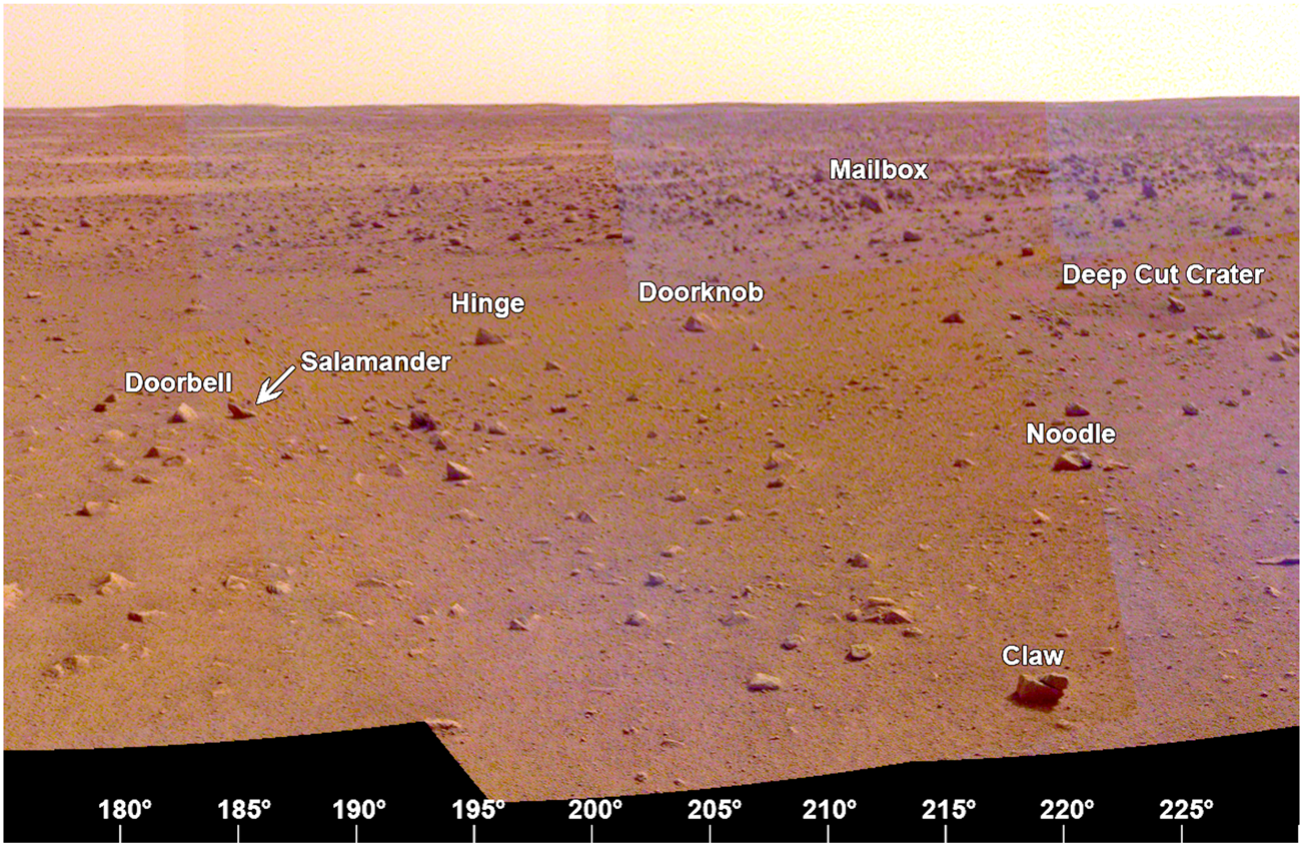
The view from the lander looking to the south‐southwest (175° to 230°) showing the rockier terrain of Homestead hollow and the rockier terrain outside the crater (e.g., Mailbox rock). Many of these rocks can also be seen in the ICC image beyond the workspace (Figure 26). A portion of the afternoon panorama that has been stretched and is not true color.

The area to the south‐southwest (180° to 225° azimuth, Figure 21) includes Rocky Field, where the hollow is covered by many small rocks. Rocks in the hollow include Doorbell at 182°, Doorknob at 205°, and Claw and Noodle at ~220°. A shallow crater (Deep Cut) with small rocks in it and around it is ~10 m away at an azimuth of 225°. Most of the rocks near the rim of Homestead hollow appear perched (Grant et al., 2020). Most of the smaller rocks in the hollow are bright, as if covered by dust.

**Figure 22:**
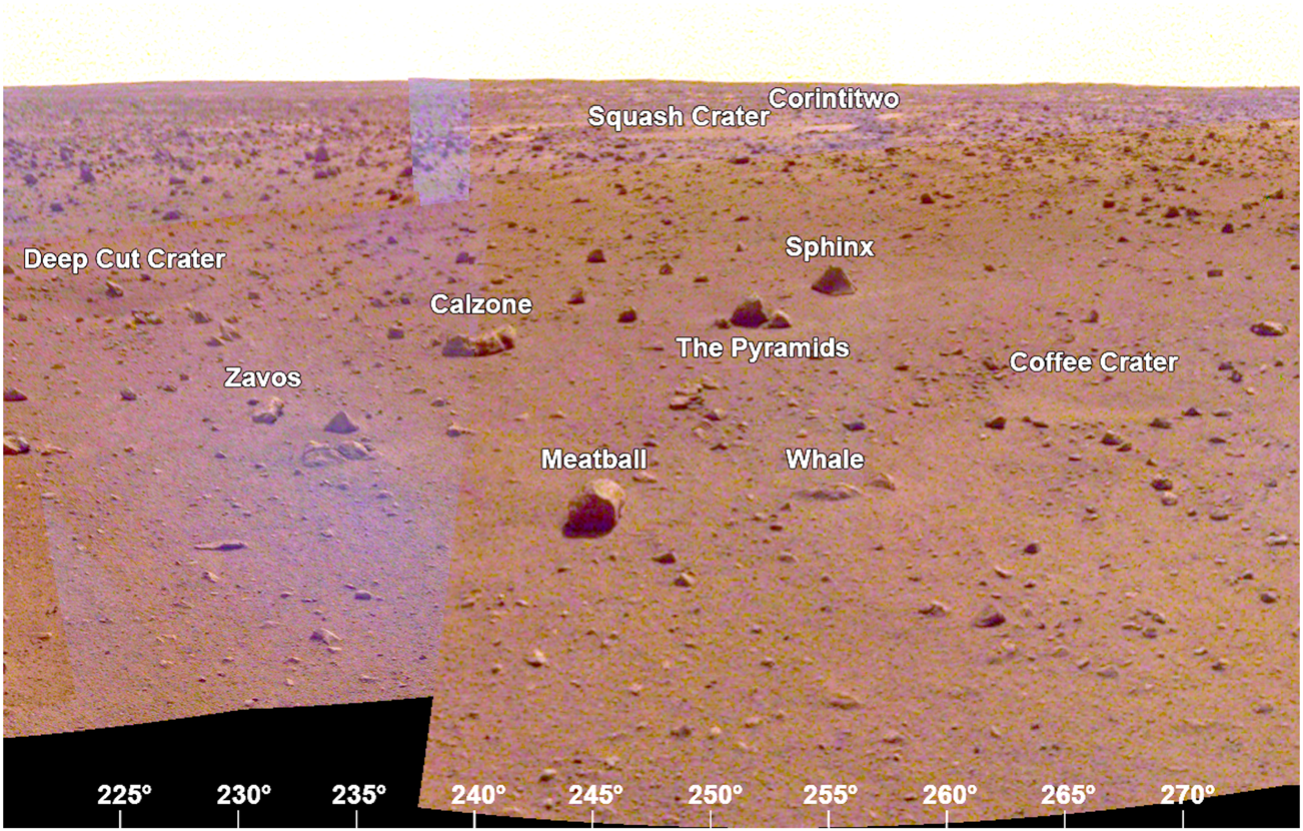
The view from the lander looking to the west‐southwest (220° to 275°) showing the rockier terrain of Homestead hollow (e.g., Calzone, Meatball, and The Pyramids) and the indistinct rim. Note Corintitwo crater (the second Corinto secondary crater in view) is about 40 m away. A portion of the afternoon panorama that has been stretched and is not true color.

The area to the west‐southwest (220° to 275° azimuth, Figure 22) includes the largest rocks in Rocky Field and Homestead hollow. Some of these rocks include Zavos at 230°, Calzone at 240°, Meatball at 245°, The Pyramids at 251°, and Sphinx and Whale at 255°. Many of these are perched and are interpreted to have been deposited as ejecta during impact of a nearby crater (Grant et al., 2020; Warner et al., 2020). The rim of the hollow in this direction is indistinct, characterized by a gentle slope. A second Corinto secondary crater, Corintitwo, 3 m diameter, is ~43 m away at an azimuth of 256°.

**Figure 23:**
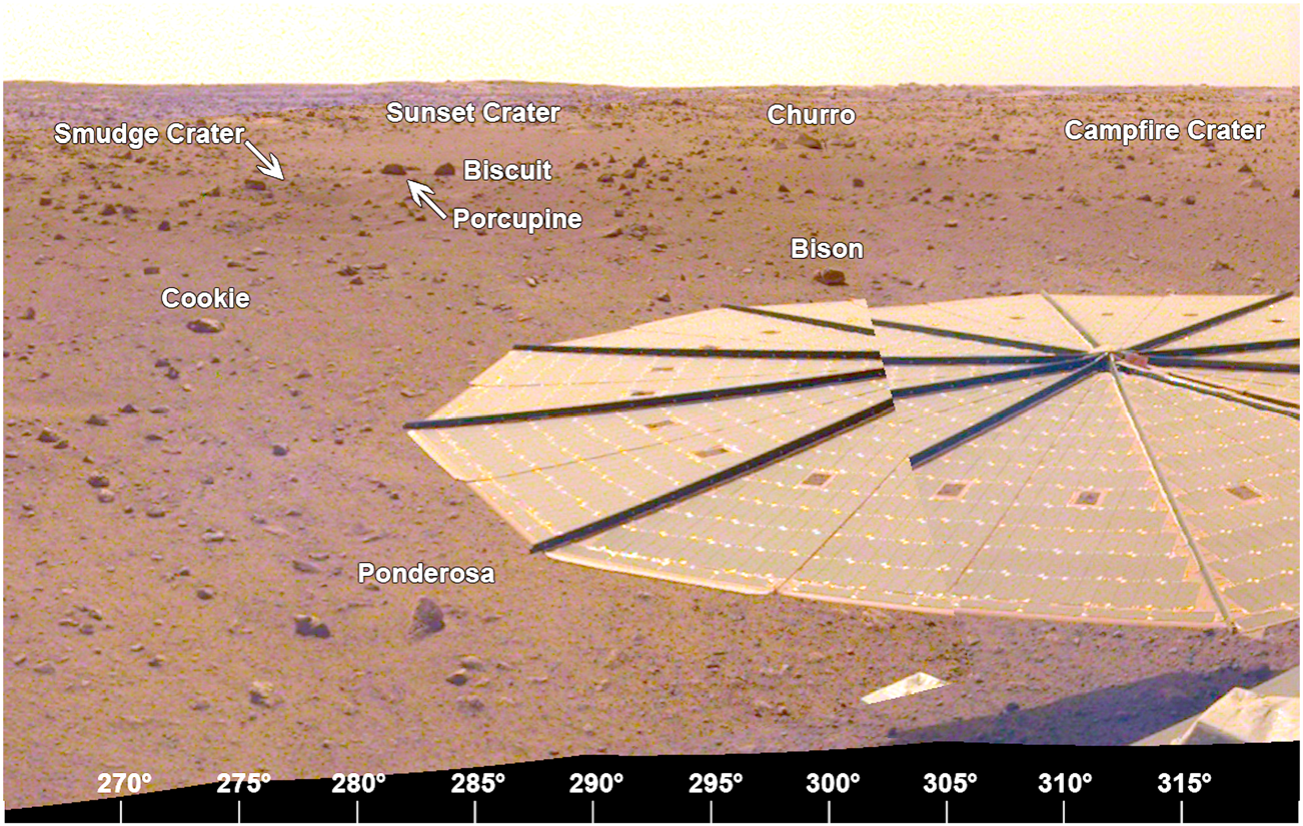
The view from the lander looking to the west‐northwest (265° to 320°) showing the −*Y* SM frame solar panel in the foreground and the rockier terrain of western Homestead hollow. Sunset crater, about 14 m away, and Smudge and Campfire craters can be seen, along with Churro rock. A portion of the afternoon panorama that has been stretched and is not true color.

The area to the west‐northwest (270° to 315° azimuth, Figure 23) shows the second solar panel (−*Y* in the SM frame) and the rockier terrain of Homestead hollow. There is a slight elevation gain beyond Homestead hollow; Sunset crater is ~14 m away and a smaller crater, Smudge, is a slightly closer. The rim of Homestead hollow here is nothing more than a shallow slope and rocks appear as plentiful inside as they are outside the hollow.

**Figure 24:**
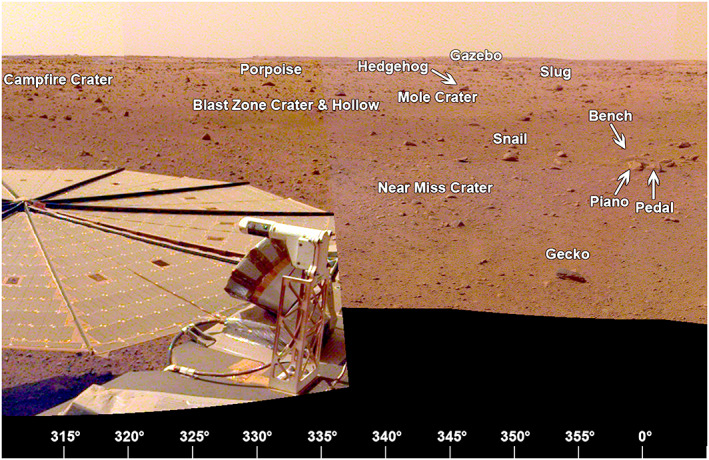
The view from the lander looking to the north‐northwest (310° to 0°) showing the −*Y* SM frame solar panel, the rockier terrain of Homestead hollow, several small craters and rocks. Note the −*Y* SM frame TWINS mast. The wind sensor is at the rounded end of the top of the mast and the temperature sensor is below (Figure 11). A portion of the afternoon panorama that has been stretched and is not true color.

The area to the north‐northwest (310° to 360° azimuth, Figure 24) includes Rocky Field with a number of rocks to the north (Gecko, Piano, Bench, Snail, and Slug). Most of them appear perched. Three small craters, Near Miss, Blast Zone, and Mole, are 5, 12, and 16 m away from the lander at 325° to 350° azimuth. All are shallow sandy depressions. The rim of Homestead hollow is ~15 m away and not very distinct with generally rockier terrain beyond.

**Figure 25:**
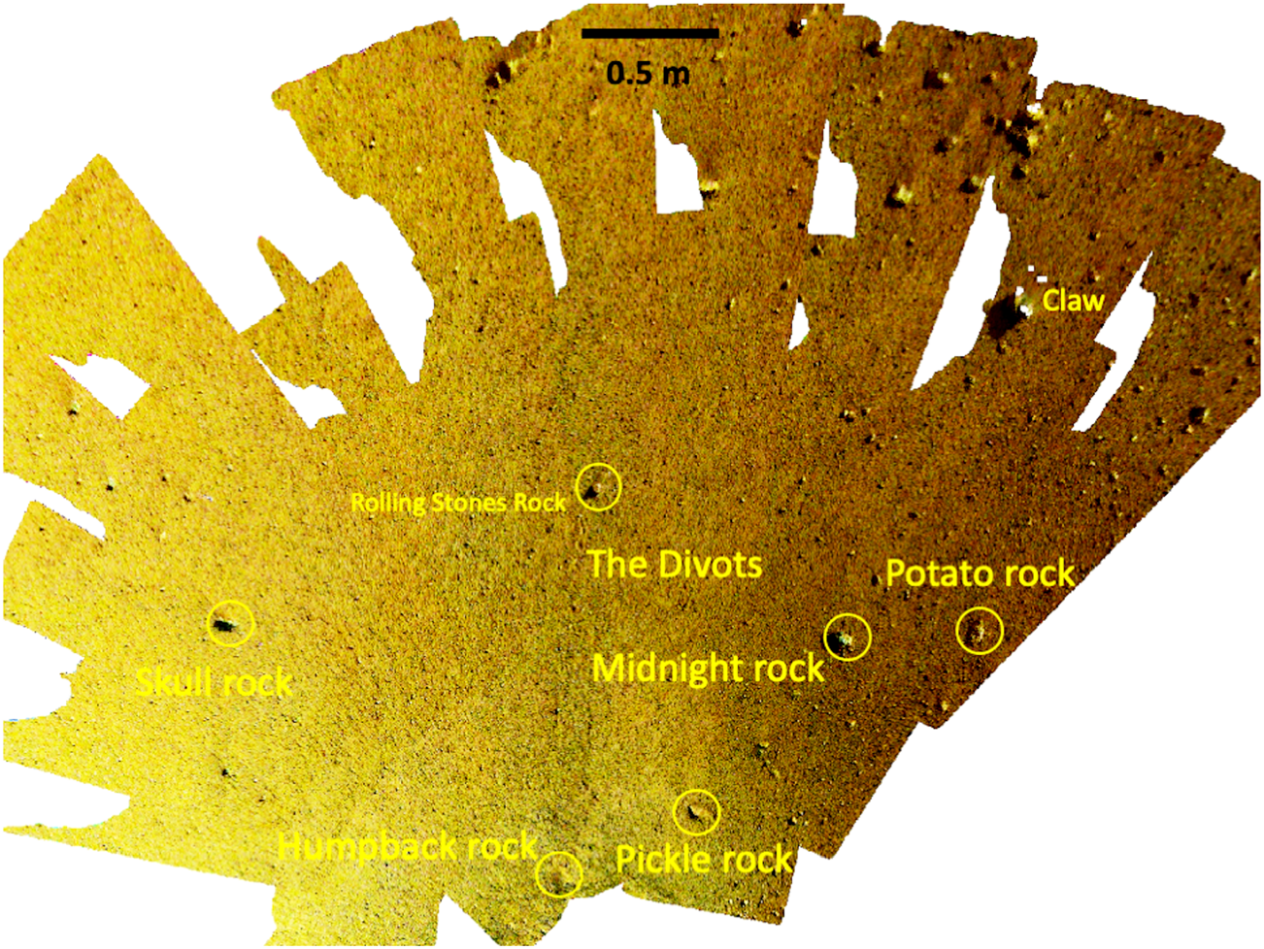
IDC image mosaic of the area including the workspace to the south of the lander (toward the top of the mosaic), showing the names of rocks.

**Figure 26:**
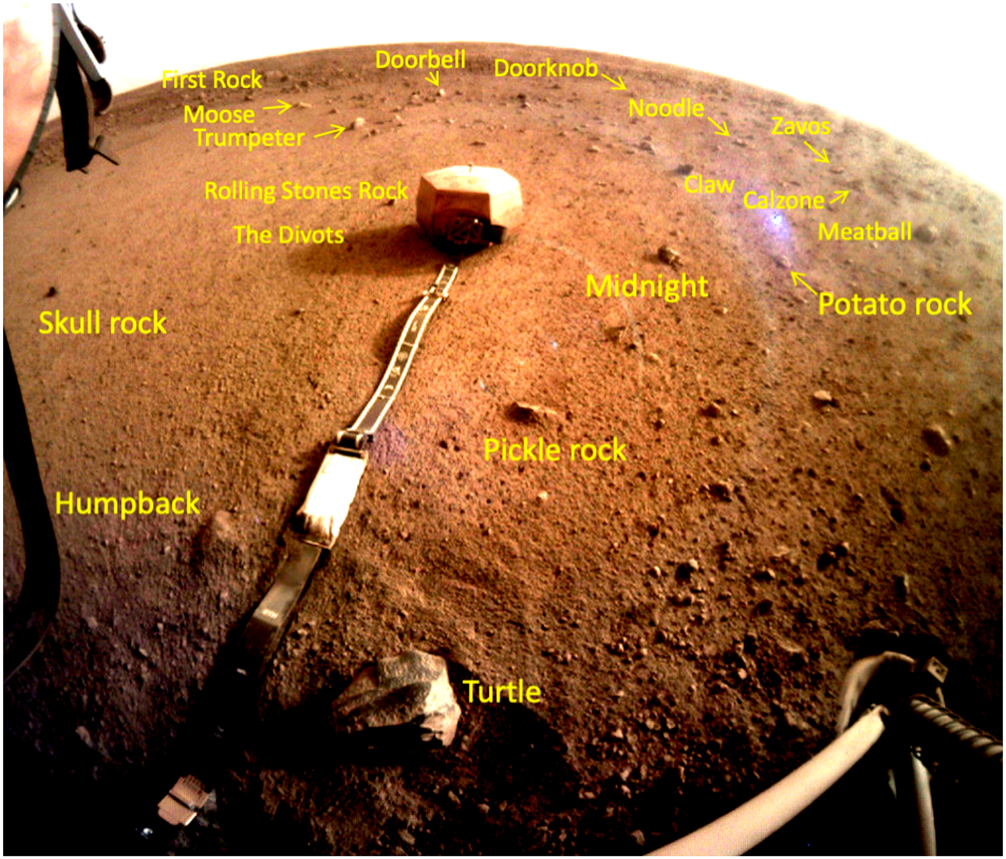
ICC image acquired after the SEIS was deployed but before the WTS, showing the rocks that have been named. Note the rocks can be correlated with those in the IDC mosaic of the workspace (Figure 25) as well as the portions of the panorama looking toward the south (Figures 20, 21, 22). The diameter of the lander footpad is 29 cm. ICC image C000M0058_601693411EDR_F0000_0461M acquired on Sol 58.

The areas to the south (south‐southeast, south‐southwest, and southwest) are those that can also be seen in the far field of the ICC images and closer in the workspace that were characterized by the IDC workspace mosaics and DEMs. The workspace is devoid of rocks larger than a few cm in diameter; however, around the workspace are a number of rocks larger than ~5 cm including Humpback, Pickle, Midnight, Potato, Claw, and Skull (Figure 25). The 5 cm diameter, Rolling Stones Rock, named by NASA, created ~10 divots when the blast from the spacecraft retro‐rockets during landing caused it to skip and hop ~1 m across the surface (Golombek, Warner, et al., 2020). The ICC images (e.g., Figure 26) also includes all of these rocks as well as First Rock, Moose, Trumpeter (observed to the south‐southeast), Doorbell, Doorknob, Claw and Noodle (observed to the south‐southwest), and Zavos, Calzone, and Meatball (observed to the southwest).

## 6 Summary and Conclusions

After landing HiRISE imaged the InSight lander on 6 December 2018 based on coordinates provided by RISE from the first day of X‐band radio tracking. This image was registered to a carefully constructed hierarchical orthoimage and topographic base map pyramid composed of 25 cm/pixel HiRISE images, 6 m/pixel CTX images, and 12.5 m/pixel HRSC images all referenced to the IAU/IAG 2000 positive east planetocentric coordinate system based on 463 m/pixel MOLA data. In this coordinate system, InSight is located at 4.50238417°N, 135.62344690°E, at an elevation of −2,613.426 m with respect to the MOLA geoid in the northwest‐central portion of the reference landing ellipse in western Elysium Planitia. The RISE location after 34 sols of tracking is ~220 m to the west, which is a measure of the uncertainty between the cartographic map and inertial coordinates in this part of Mars, which is important for accurately landing spacecraft. The InSight heatshield is located 0.762 km downtrack (to the east), and the backshell/parachute is located 0.553 km to the southeast.

IDC stereo image mosaics and DEMs made from them became available on 10 and 14 December 2018. Two mosaics at 2 and 1 mm per elevation posting showed the workspace to be particularly benign with a sandy, granule and pebbly surface, few rocks, and low slopes that met all of the instrument deployment requirements over most of the deployment area. After the locations for the instruments were selected, the testbed was sculpted to match the workspace on Mars. This enabled tests of deployment of the instruments to the selected locations using an engineering model lander and arm. Instrument placement locations were certified and approved by the project on 17 December 2018.

On Mars, the instruments were placed in the workspace by the arm using coordinates in the Instrument Deployment Arm frame, whose origin is the shoulder joint of the arm on the lander deck. The IDA frame can be translated into the spacecraft mechanical frame as well as the site frame, which has *Z* along the gravity vector, with *X* north and *Y* east (right hand rule). The site frame has the same origin as the IDA frame but accounts for the local tilt and orientation of the spacecraft provided by the IMU. Because HiRISE easily resolves the spacecraft and the large circular solar panels, its center can be related to the spacecraft mechanical frame. Offsetting the IDC orthophoto of the workspace in the HiRISE image yields the location of the instruments in the cartographic frame as well as the center of lander deck and the origin of the IDA and site frames. The locations of the SEIS, HP^3^, magnetometer (including orientation), pressure sensor, temperature sensors, wind sensors, and RISE antennas are all determined by translating between the spacecraft mechanical, IDA, and cartographic frames.

A viewshed from a height of 1.5 m above the lander location was created from the HiRISE DEM and shows the lander is located on a broad gentle slope down to the east in which crater rims 400 m and 2.4 km away can be distinguished. A slope to the north limits the horizon to ~50 m away formed by a degraded impact crater with three rocks and an eolian bedform. The backshell and parachute are in a slight depression and are not visible from the lander. The heatshield is in an area that should be in view, but at its distance, its maximum height would only be ~1 pixel high. Azimuths to rocks, craters, and eolian bedforms observed in controlled lander panoramas (in site frame) and in carefully georeferenced HiRISE images match within 1°, indicating that the azimuth and tilt recorded by the IMU used to define the site frame are accurate.

InSight landed in a quasi‐circular depression, interpreted to be a degraded impact crater called Homestead hollow with a sandy, granule, and pebble rich surface. The western portion of the hollow is rockier (Rocky Field) as is the region beyond the hollow. Rocks in the hollow and at the edge of the hollow appear perched and many are tabular and angular. Individual craters, rocks and eolian bedforms in the controlled lander panoramas have been identified, named, and mapped onto a HiRISE image. Fifteen small craters between 1 and 10 m diameter and 5 and 42 m from the lander have been identified in both InSight and HiRISE images, with their distances, azimuths, and diameters recorded. All craters are shallow sandy depressions. Two secondary craters with distinctive bright ejecta seen in HiRISE images are interpreted to be from the fresh, rayed crater Corinto located ~800 km to the northeast. These secondaries are omnipresent across the landing ellipse. Eolian bedforms are located near the rims of relatively fresh, distant (50 and 400 m diameter) impact craters. Distant shallow, bright quasi‐circular depressions interpreted to be degraded and filled impact craters (hollows) are common toward the east. This detailed information about the location of the InSight lander, its instruments, and setting are important for interpretations of the geophysical and environmental sensors data that have been returned and will continue to be returned from the InSight lander.

## Data Availability

All InSight image data discussed in this paper are in the Planetary Data System Geosciences node (https://pds‐geosciences.wustl.edu/missions/insight/index.htm). All other Mars imaging data are in the Cartography and Imaging Node (https://pds‐imaging.jpl.nasa.gov/). The Context Camera orthoimage and DEM in which the lander is located are available at https://astrogeology.usgs.gov/search/map/Mars/InSight/landing_site/F04_037262_1841_XN_04N224W_6m_ORTHO (Fergason et al., 2017). The HiRISE orthoimage and DEM in which the lander is located are available at https://www.uahirise.org/dtm/dtm.php?ID=ESP_037262_1845 (Fergason et al., 2017), and other HiRISE images acquired are also available at https://hirise.lpl.arizona.edu/ website. The HRSC orthoimage where the InSight lander is located produced by Golombek et al. (2017) is included in Golombek (2020). The CTX and HiRISE orthoimages and DEMS produced by Fergason et al. (2017) in which the lander is located (above), which have been georeferenced as described in the text, are also available in Golombek (2020). The viewsheds in Figures 13 and 14 produced from the HiRISE DEM are included in Golombek (2020). Finally, the morning, midday (afternoon) and evening InSight IDC panoramas used to create Figures 17–24 are also available in Golombek (2020).
